# Analogs of the Frog-skin Antimicrobial Peptide Temporin 1Tb Exhibit a Wider Spectrum of Activity and a Stronger Antibiofilm Potential as Compared to the Parental Peptide

**DOI:** 10.3389/fchem.2017.00024

**Published:** 2017-04-11

**Authors:** Lucia Grassi, Giuseppantonio Maisetta, Giuseppe Maccari, Semih Esin, Giovanna Batoni

**Affiliations:** ^1^Department of Translational Research and new Technologies in Medicine and Surgery, University of PisaPisa, Italy; ^2^Center for Nanotechnology Innovation @NEST, Italian Institute of TechnologyPisa, Italy

**Keywords:** Temporin 1Tb, analogs, biofilm, peptide design, antimicrobial peptides

## Abstract

The frog skin-derived peptide Temporin 1Tb (TB) has gained increasing attention as novel antimicrobial agent for the treatment of antibiotic-resistant and/or biofilm-mediated infections. Nevertheless, such a peptide possesses a preferential spectrum of action against Gram-positive bacteria. In order to improve the therapeutic potential of TB, the present study evaluated the antibacterial and antibiofilm activities of two TB analogs against medically relevant bacterial species. Of the two analogs, TB_KKG6A has been previously described in the literature, while TB_L1FK is a new analog designed by us through statistical-based computational strategies. Both TB analogs displayed a faster and stronger bactericidal activity than the parental peptide, especially against Gram-negative bacteria in planktonic form. Differently from the parental peptide, TB_KKG6A and TB_L1FK were able to inhibit the formation of *Staphylococcus aureus* biofilms by more than 50% at 12 μM, while only TB_KKG6A prevented the formation of *Pseudomonas aeruginosa* biofilms at 24 μM. A marked antibiofilm activity against preformed biofilms of both bacterial species was observed for the two TB analogs when used in combination with EDTA. Analysis of synergism at the cellular level suggested that the antibiofilm activity exerted by the peptide-EDTA combinations against mature biofilms might be due mainly to a disaggregating effect on the extracellular matrix in the case of *S. aureus*, and to a direct activity on biofilm-embedded cells in the case of *P. aeruginosa*. Both analogs displayed a low hemolytic effect at the active concentrations and, overall, TB_L1FK resulted less cytotoxic toward mammalian cells. Collectively, the results obtained demonstrated that subtle changes in the primary sequence of TB may provide TB analogs that, used alone or in combination with adjuvant molecules such as EDTA, exhibit promising features against both planktonic and biofilm cells of medically relevant bacteria.

## Introduction

The development and rapid spread of antibiotic resistance among clinically relevant bacteria has dramatically reduced the effectiveness of antimicrobial therapies, thereby emerging as a major challenge for modern medicine (Boucher et al., 2009; Högberg et al., 2010). The ability of bacteria to form biofilms, architecturally complex cell aggregates embedded in an extracellular polymeric substance (EPS) and intrinsically tolerant to conventional antibiotics, further exacerbates the problem of bacterial resistance and is responsible for the persistence and chronicization of many types of infections (Costerton et al., 1999). Biofilms can be up to 1,000-fold more resistant to antimicrobial agents than their planktonic counterparts thanks to unique phenotypic and metabolic properties that allow them to implement resistance mechanisms at the community level. These include the presence of the EPS that reduces the diffusion of antibacterial compounds into the biofilm structure, the overall low growth rate of biofilm-forming bacteria, the presence of subpopulations of cells in a dormant state (“persisters”), and the cell proximity that promotes the horizontal gene transfer and the acquisition of mobile genetic elements encoding resistance (Høiby et al., 2010; Batoni et al., 2016a).

Over the last years, antimicrobial peptides (AMPs) have gained increasing attention as novel antimicrobial drugs for the control of infections sustained by antibiotic-resistant bacteria and/or bacterial biofilms. Due to their main mechanism of action, which involves the disruption of cell membrane integrity, AMPs exert a strong antimicrobial activity against a broad spectrum of pathogens, including multidrug-resistant bacterial strains, and generally prove a low frequency in inducing resistance (Zasloff, 2002). Moreover, they are able to target metabolically inactive and even non-growing cells that are commonly found within microbial biofilms (Di Luca et al., 2014; Batoni et al., 2016a). To date, over 2500 AMPs have been identified and evaluated for their antimicrobial activity (Antimicrobial Peptide Database: aps.unmc.edu/AP/main.php) and a growing number of them have also been tested against biofilms (BaAMPs database: www.baamps.it) (Di Luca et al., 2015).

The frog skin-derived peptide temporin 1Tb (TB) is considered a promising template for the development of next-generation antibiotics (Di Grazia et al., 2014). It is a 13-amino acid, mildly cationic (net charge +2) and α-helical peptide endowed with a bacterial membrane-perturbing activity (Mangoni et al., 2000). The peptide has previously demonstrated a fast and potent bactericidal action particularly against Gram-positive bacterial species, such as multidrug-resistant nosocomial strains of *Staphylococcus aureus* and *Enterococcus faecium* (Mangoni et al., 2008). The antibiofilm properties of TB have been also investigated showing high activity against both forming and mature biofilms of *Staphylococcus epidermidis*, especially when the peptide was used in combination with EDTA (Maisetta et al., 2016). Interestingly, it has been recently reported that the peptide is able to penetrate eukaryotic cells, kill intracellular *S. aureus* and promote wound-healing, further important properties in view of a therapeutic development (Di Grazia et al., 2014). Despite the many favorable features of TB, the preferential spectrum of activity of the peptide against Gram-positive bacteria partially limits its translatability into a clinically useful agent. The rational *in silico* design of novel peptides with optimized structural properties and the chemical manipulation of existing ones represent valid approaches to overcome the limitations of native peptides (Maccari et al., 2013). The introduction of appropriate changes in the peptide primary sequence and, thus, the alteration of crucial physicochemical parameters of AMPs (e.g., cationicity, hydrophobicity and amphipaticity) may significantly influence their bactericidal, cytotoxic and antibiofilm potential allowing to obtain molecules with improved antimicrobial efficacy and broader spectrum of action (Conlon et al., 2007; Takahashi et al., 2010; Batoni et al., 2016b). The aim of the present study was the optimization of TB activity against both planktonic bacteria and biofilms of medically relevant bacterial species. In particular, the antibacterial, antibiofilm and cytotoxic properties of TB were compared with those of two recently developed TB analogs. The first one (TB_KKG6A), described by Avitabile and co-workers, was initially obtained by Ala scanning on TB sequence and further optimized by increasing its positive charge (Avitabile et al., 2013). TB_KKG6A was found to efficiently interact with the lipopolysaccharide (LPS) of the Gram-negative bacterium *Escherichia coli* and to fold upon binding into a bent helix (Malgieri et al., 2015). The second one (TB_L1FK), firstly described in this study, was designed by us through statistical-based computational strategies (Maccari et al., 2013). Overall, TB analogs displayed a faster and stronger bactericidal activity than the parental peptide, especially against Gram-negative bacterial species in planktonic form. In addition, a marked antibiofilm activity against preformed biofilms of *S. aureus* and *Pseudomonas aeruginosa* was observed for both TB_KKG6A and TB_L1FK used in combination with EDTA, highlighting the potential of combinatorial drug therapies in the management of biofilm-related infections. When assayed on mammalian cells, TB_L1FK showed a lower cytotoxic activity against human epithelial cells as compared to TB_KKG6A, emerging as a promising molecule for the topical treatment of biofilm-associated infections.

## Materials and methods

### Peptides

TB, TB_L1FK (designed as reported in “Results”) and TB_KKG6A were synthesized by Proteogenix (Schiltigheim, France). Analysis of the synthetic peptides by high performance chromatography (HPLC) and mass spectrometry revealed purity over 98%. Peptides were diluted in milli-Q water to obtain a stock solution of 1 mM and stored at −80°C. The main features of the peptides are shown in Table 1.

**Table 1:** **Main structural and physicochemical features of the peptides used in the study**.

**Peptide**	**Sequence**	**Molecular weight**	**Charge**	**Hydrophobicity^a^**
TB	LLPIVGNLLKSLL-NH_2_	1392.78	+2	3.62
TB_L1FK	**F**LPIVGLLKSLL**K**-NH_2_	1440.86	+3	3.43
TB_KKG6A	**KK**LLPIV**A**NLLKSLL-NH_2_	1663.15	+4	1.91

a *Hydrophobicity was calculated with the combined consensus scale (CCS) through the BaAMPs database (Di Luca et al., 2015)*.

### EDTA

Disodium ethylenediaminetetraacetic acid (EDTA) was purchased from Sigma-Aldrich (St. Louis, USA). A stock solution of EDTA (0.5 M) was prepared in milli-Q water by adjusting the pH to 8.0 with NaOH. The working solution (50 mM) was obtained by diluting the stock solution in milli-Q water, sterile filtered and stored at 4°C.

### Bacterial strains and culture conditions

The reference laboratory strains *Klebsiella pneumoniae* (ATCC BAA-1706), *P. aeruginosa* (ATCC 27853), *S. aureus* (ATCC 33591), and *S. epidermidis* (ATCC 35984) were used for the study. For the preparation of stock cultures, bacterial strains were grown in Tryptone Soy Broth (TSB) (Oxoid, Basingstoke, UK) until mid-log phase, subdivided in aliquots and stored at −80°C. For the colony-forming units (CFU) count, serially diluted bacterial suspensions were plated on Tryptone Soy Agar (TSA) (Oxoid) and incubated for 24 h at 37°C.

### Bactericidal activity and killing kinetics in sodium-phosphate buffer

The bactericidal activity of TB, TB_L1FK and TB_KKG6A against *K. pneumoniae, P. aeruginosa, S. aureus*, and *S. epidermidis* was evaluated by the microdilution method in sodium-phosphate buffer (10 mM SPB, pH 7.4). Bacterial strains were grown in TSB until exponential phase and suspended in SPB to reach a density of 1 × 10^7^ CFU/mL. A volume of 10 μL of the bacterial suspensions was added to 90 μL of SPB containing different concentrations of the peptides (from 1.5 to 48 μM). Bacteria suspended in SPB alone were used as cell viability control. Samples were incubated at 37°C with shaking for various times (5, 15, 30, 60, and 90 min), subsequently diluted 10-fold in TSB and plated on TSA to determine the number of CFU. The minimal bactericidal concentration (MBC) was defined as the minimal concentration of peptide causing a reduction of at least 3 Log_10_ in the number of viable bacteria after 90 min of incubation (Mangoni et al., 2008).

### Biofilm inhibition assay

The ability of TB, TB_L1FK, and TB_KKG6A to prevent biofilm formation was evaluated against *S. aureus* and *P. aeruginosa*. Bacteria were grown overnight in TSB/Glc (TSB added with 0.25% (v/v) glucose) at 37°C. Stationary-phase cultures were diluted 1:1,000 in Biofilm Promoting Medium (BPM; TSB diluted 1:1 with 10 mM SPB at pH 7.4 and supplemented with 0.25% glucose). Bacterial suspensions were inoculated into flat-bottom polystyrene 96-well microplates (Corning Costar, Lowell, USA), in the absence (negative control) or in the presence of different concentrations of each peptide (from 12 to 48 μM). Microplates were incubated statically at 37°C for 24 h and biofilm biomass was estimated by crystal violet (CV) staining assay. To this aim, biofilms were rinsed three times with phosphate-buffer saline (PBS), air-dried for 15 min and incubated with 0.1% (w/v) CV (bioMérieux, Florence, Italy) for 15 min. The excess of CV was removed by washing the plates with PBS, while biofilm-associated CV was extracted with 98% ethanol (Sigma Aldrich) and quantified by measuring the optical density at 570 nm (OD_570_) in a microplate reader (Model 550, Bio-Rad Laboratories Srl, Italy).

### Biofilm treatment assay

The activity of TB, TB_L1FK, and TB_KKG6A against preformed (24-h old) biofilms of *S. aureus* and *P. aeruginosa* was also investigated. Briefly, biofilms were allowed to form for 24 h in flat-bottom 96-well microplates in the absence of antimicrobial compounds. Established biofilms were then washed three times with PBS in order to remove non-adherent cells and incubated in fresh BPM with different concentrations of the three peptides (from 15 to 120 μM). After 24 h of incubation, the viability of biofilm-associated cells was evaluated by CFU counting. For this purpose, biofilms were washed three times with PBS and bacterial cells were detached from the surface of the wells with a pipette tip, vigorously vortexed and plated in serial dilutions on TSA.

### Evaluation of the synergistic effect between TB analogs and EDTA on preformed biofilms

TB_L1FK and TB_KKG6A were combined with EDTA in order to enhance their activity against preformed biofilms of *S. aureus* and *P. aeruginosa*. To this aim, 24 h-old biofilms of the two bacterial species were exposed to different concentrations of the peptides (15 and 30 μM), alone and in combination with EDTA (1.25 and 2.5 mM). Microplates were incubated statically at 37°C for 24 h. Following incubation, the antibiofilm effect was evaluated in terms of number of biofilm-associated viable cells as previously described.

### Evaluation of the synergistic effect between TB analogs and EDTA on planktonic bacteria in biofilm-like conditions

The antibacterial activity of TB_L1FK and TB_KKG6A, used alone and in combination with EDTA, was also tested against planktonic cells of *S. aureus* and *P. aeruginosa*. The Minimal Inhibitory Concentration (MIC) of the peptides, EDTA and the peptide-EDTA combinations was determined by the microdilution method under the same experimental conditions used for the biofilm assay. Briefly, bacteria from overnight cultures were diluted 1:1,000 in BPM and incubated for 24 h at 37°C in propylene tubes in the presence of TB_L1FK and TB_KKG6A (from 3.75 to 120 μM), alone and combined with EDTA (from 0.3 to 10 mM). MIC was defined as the lowest concentration of the compounds resulting in the complete inhibition of visible growth. The effect of each combination on cell growth was studied using an adapted Fractional Inhibitory Concentration (FIC) index analysis. FIC index was calculated as follows: Σ (FIC_A_ + FIC_B_), where FIC_A_ is the MIC of compound A in combination/MIC of compound A alone, and FIC_B_ is the MIC of compound B in combination/MIC of compound B alone. Synergism was defined as a FIC index ≤ 0.5, indifference as a FIC index > 0.5 and antagonism as a FIC index > 4 (Katragkou et al., 2015; Dosler et al., 2016).

### Hemolysis assay

Hemolytic activity of TB and its analogs was tested against human red blood cells (RBCs) as previously described (Tavanti et al., 2011). Briefly, peripheral blood obtained from healthy donors was centrifuged (1,000 × g for 10 min, 4°C) and washed three times with PBS (Euroclone, Milan, Italy). A suspension of RBCs (4%, v/v) was mixed with various concentrations of the peptides (from 12 to 96 μM) into a round-bottom polystyrene 96-well microplate (Corning Costar). RBCs suspended in PBS alone were used as negative control (0% hemolysis), while cells lysed with 0.1% Triton X-100 were taken as positive control (100% hemolysis). The microplate was incubated for 1 h at 37°C and then centrifuged at 1,000 × g for 20 min, 4°C. Supernatants were transferred to a new plate and the optical density at 450 nm (OD_450_) was measured by means of a microplate reader. The hemolytic activity was quantified according to the following formula: hemolysis (%) = [(OD _peptide_ – OD _negative control_)/(OD _positive control_ – OD _negative control_)] × 100.

### Cytotoxicity assay

Cytotoxic activity of the peptides was assessed against human peripheral blood mononuclear cells (PBMCs) and human non-small-cell lung adenocarcinoma A549 cells (ATCC CCL-185).

PBMCs were isolated from buffy coats by conventional density gradient centrifugation. For this purpose, buffy coats were diluted 1:1 in PBS supplemented with 10% (v/v) sodium citrate (Sigma-Aldrich) and layered on Lympholyte-H gradient medium (Euroclone). Following centrifugation at 200 × g for 20 min at room temperature, the supernatant was eliminated in order to remove platelets. Buffy coats were further centrifuged at 800 × g for 20 min at room temperature and the lymphocyte/monocyte layer was harvested at the sample/medium interface. PBMCs were washed three times with PBS containing 0.5% (wt/v) bovine serum albumin (BSA; Sigma-Aldrich) and 10% sodium citrate, counted and re-suspended in RPMI 1640 (Euroclone) added with 10% (v/v) fetal calf serum (FCS; Euroclone) and 2 mM L-glutamine. Cells (1 × 10^5^ per well) were seeded into round-bottom 96-well microplates (Corning Costar) and incubated with increasing concentrations of the peptides (from 12 to 96 μM) for 24 h at 37°C, 5% CO_2_. PBMCs incubated with culture medium were used as negative (cell viability) control, while cells treated with cycloheximide (2 mg/mL) served as a positive (death) control.

A549 cells were grown in tissue culture flasks in Dulbecco's modified Eagle's medium (DMEM; Euroclone) containing 10% FCS and 2 mM L-glutamine. Confluent monolayers of A549 cells were washed with PBS, treated with a trypsin-EDTA solution (Sigma-Aldrich), centrifuged at 300 × g for 10 min, counted and re-suspended in complete DMEM at a final density of 5 × 10^4^ cells/mL. A volume of 200 μL of the cell suspension was seeded into flat-bottom 96-well microplates (Corning Costar) and cultured for 24 h at 37°C, 5% CO_2_. Peptides at a final concentration of 12–96 μM were added to the cells and incubated for further 24 h at 37°C, 5% CO_2_. A549 cells incubated with culture medium were used as negative (cell viability) control, while cells treated with cycloheximide (2 mg/mL) served as a positive (death) control.

Cytotoxic activity was evaluated by the propidium iodide (PI) flow cytometric assay. To this end, PBMCs were washed once in PBS, resuspended in 100 μL, and incubated with 5 μL of a PI solution (50 μg/mL) (Sigma-Aldrich) for 4 min in the dark. Similarly, A549 cells were harvested by trypsinization, rinsed once with PBS and exposed to PI. Counting of viable (PI-negative) and dead (PI-positive) cells was carried out with a BD Accuri C6 flow cytometer (BD Biosciences, Mountain View, CA) and data were analyzed using BD Accuri C6 software (BD Biosciences). Cytotoxic effect was determined according to the following formula: Cytotoxicity (%) = [(PI-positive cells _peptide_ – PI-positive cells _negative control_)/(100 – PI-positive cells _negative control_)] × 100. The IC_50_ values (Inhibitory Concentration) were defined as the concentration of the peptides causing 50% cell death as compared to the untreated control.

### Statistical analysis

All the experiments were performed at least in triplicate, unless otherwise specified. Differences between mean values of groups were evaluated by one-way analysis of variance (ANOVA) followed by Tukey-Kramer *post-hoc* test, after normalization of the data. A *p*-value < 0.05 was considered statistically significant.

## Results

### TB_L1FK design

In order to improve the therapeutic potential of TB, a novel peptide was computationally designed starting from TB sequence. In a previous work, chemophysical analysis of known AMPs sequences was successfully employed to design a statistical model of membrane-disrupting peptides able to account for non-natural amino acids (Maccari et al., 2013). In this work, an additional statistical model was designed to account for peptides' cytotoxic effect. Together with the previously described models for the secondary structure and the antimicrobial activity, a forth constraint was imposed in order to retain as much as possible the sequence similarity with TB. A dataset of peptides with proved cytotoxic effect was appositely designed by collecting and combining data from different bioactive peptide databases (Gupta et al., 2013). Furthermore, another set of peptides was designed to represent non-cytotoxic peptides, allowing the statistical model to grasp the features that distinguish the two sets (see Section 1.1 in the Supplementary Material). A number of filters aimed to normalize and uniform the training set of peptides were applied and then, sequences were encoded into physicochemical variables representing global and topological properties of peptides. A machine learning algorithm was adopted to build a prediction engine able to discern between toxic and non-toxic peptides (see the Supplementary Material for details in model training and validation). Model performance was evaluated by the Mathews Correlation Coefficient (MCC), which assesses the prediction in terms of true and false positives and negatives. In the final configuration, a prediction model with an MCC value of 0.82 was obtained. The candidate sequence, named TB_L1FK, was designed by applying the statistical model to a particular class of Genetic Algorithms, called Multi-Objective Evolutional Algorithms (MOEA) that allows to screen for candidates that simultaneously satisfy different criterions.

**Figure 1:**
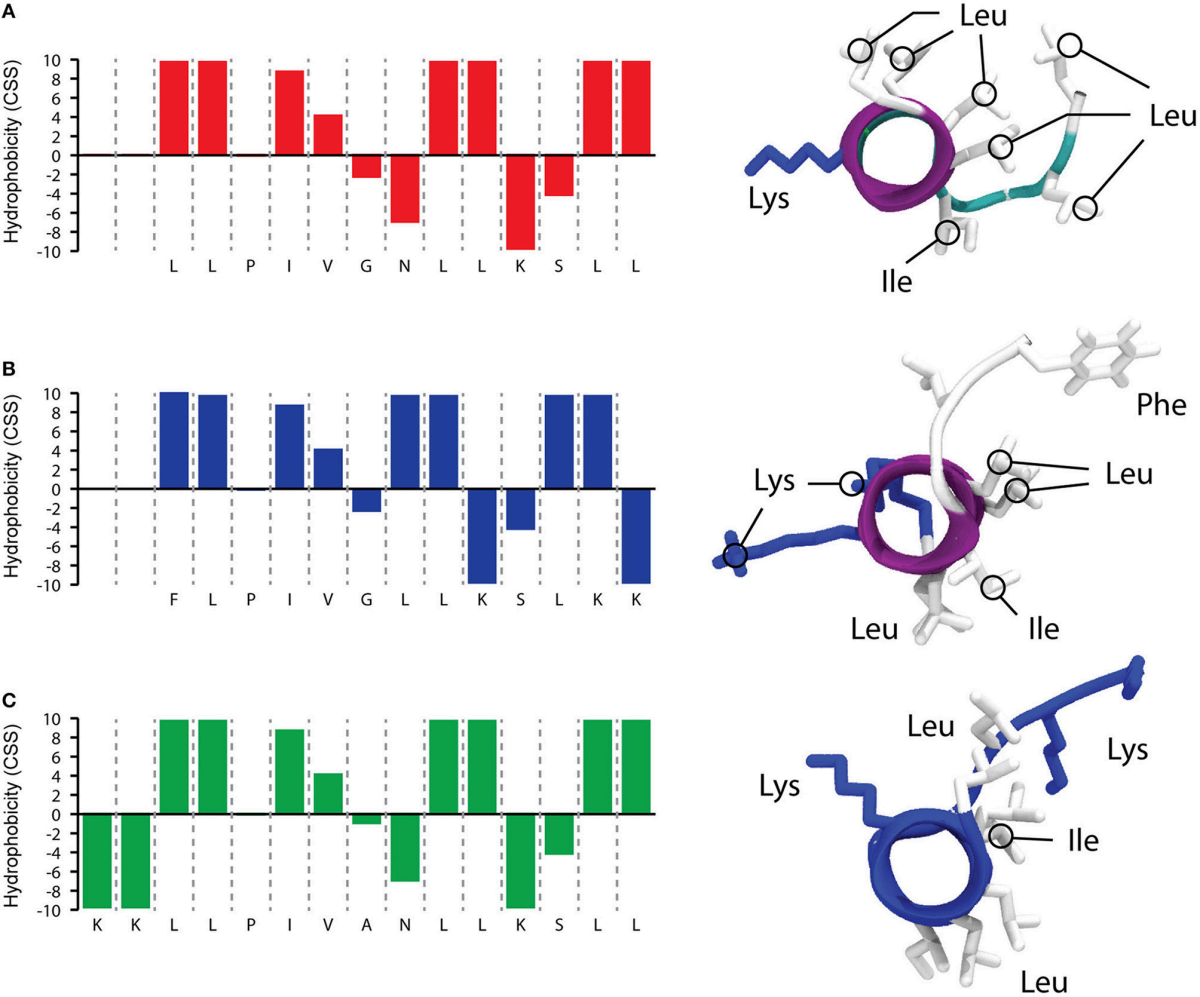
**Predictive simulations of hydrophobic profile and 3D structure of TB (A)**, TB_L1FK **(B)**, and TB_KKG6A **(C**). The hydrophobic profile calculated with the combined consensus scale (CCS) is schematized on the left side (Maccari et al., 2013). 3D structures (right side of the figure) were calculated with PEP-FOLD3 (Lamiable et al., 2016); the distribution of hydrophobic and charged residues is highlighted.

As reported in Figure 1, that shows a predictive simulation of the structure of TB and its two analogs, TB_L1FK displays similar physicochemical characteristics to the parental peptide. Hydrophobicity and net charge of TB_L1FK are close to those of TB, while TB_KKG6A presents a different hydrophobic profile and an increased net charge, particularly localized at the C-terminus. One of the aims in the computational design of TB_L1FK was to retain all the features that could infer in the membrane interaction of the peptide with the target cells. Besides, molecular hydrophobicity and net charge, as well as size and molecular weight, represent important aspects for the loading and the controlled release of peptides such as TB from nanostructured delivery systems (Piras et al., 2015).

### Bactericidal activity and killing kinetics of peptides in sodium-phosphate buffer

The antimicrobial activity of TB, TB_KKG6A, and TB_L1FK was evaluated in terms of MBC values toward *S. aureus* and *S. epidermidis* as models of Gram-positive bacteria and against *K. pneumoniae* and *P. aeruginosa* as models of Gram-negative bacteria. As shown in Table 2, TB was mainly active against Gram-positive bacteria and exhibited a bactericidal effect against Gram-negative bacteria only at 48 μM. Both analogs displayed a markedly increased activity compared to the parental peptide against all the bacterial species tested, but especially against the Gram-negative ones. In particular, a 2- to 8-fold reduction in the MBC compared to TB was observed against the Gram-positive bacteria, while an up to 16-fold decrease in the MBC value was observed in the case of the Gram-negative bacteria.

**Table 2:** **MBCs of TB, TB_L1FK, and TB_KKG6A against Gram-positive and Gram-negative bacteria in sodium-phosphate buffer (10 mM SPB, pH 7.4)**.

	**Gram-positive**		**Gram-negative**	
	***S. aureus* ATCC 33591**	***S. epidermidis* ATCC 35984**	***K. pneumoniae* ATCC BAA-1706**	***P. aeruginosa* ATCC 27853**
TB	12^a^	6	48	48
TB_L1FK	6	1.5	6	6
TB_KKG6A	1.5	1.5	3	3

a *Numbers represent the MBC values expressed in μM*.

**Figure 2:**
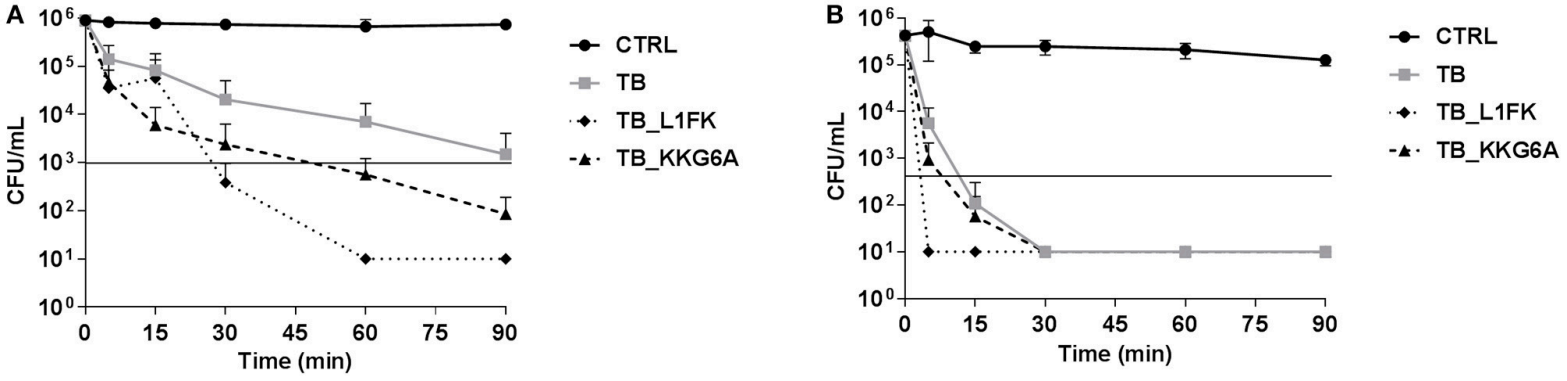
**Killing kinetics of TB, TB_L1FK, and TB_KKG6A against *S. aureus* ATCC 33591 (A)** and *P. aeruginosa* ATCC 27853 **(B)**. Bacteria were incubated in sodium-phosphate buffer (10 mM SPB, pH 7.4) with the peptides at concentrations equal to their MBCs for various times. Control (CTRL) represents untreated bacteria. Solid line indicates a reduction of ≥3 Log_10_ in the number of control bacteria at each time of incubation. A number of 10 CFU/mL was taken as detection limit. Data are expressed as mean ± standard error of at least three independent experiments.

Time-kill studies on two representative bacterial species, *S. aureus* and *P. aeruginosa*, were carried out using the peptides at concentrations equal to their MBC. TB exerted its bactericidal activity toward *S. aureus* after approximately 90 min of incubation (Figure 2A). Both TB_L1FK and TB_KKG6A exhibited a faster killing kinetics than TB against the same bacterial species causing a reduction of at least 3 Log_10_ in the number of viable bacteria within 30 and 60 min, respectively (Figure 2A). All three peptides showed a more rapid bactericidal effect against *P. aeruginosa* than against *S. aureus* (Figure 2B). In particular, TB and TB_KKG6A showed similar killing kinetics, being bactericidal after 15 min of incubation, while the most rapid bactericidal effect was exerted by TB_L1FK that determined the complete eradication of the starting bacterial inoculum within as little as 5 min of incubation (Figure 2B).

### Effect of TB and TB analogs on forming and preformed biofilms

**Figure 3:**
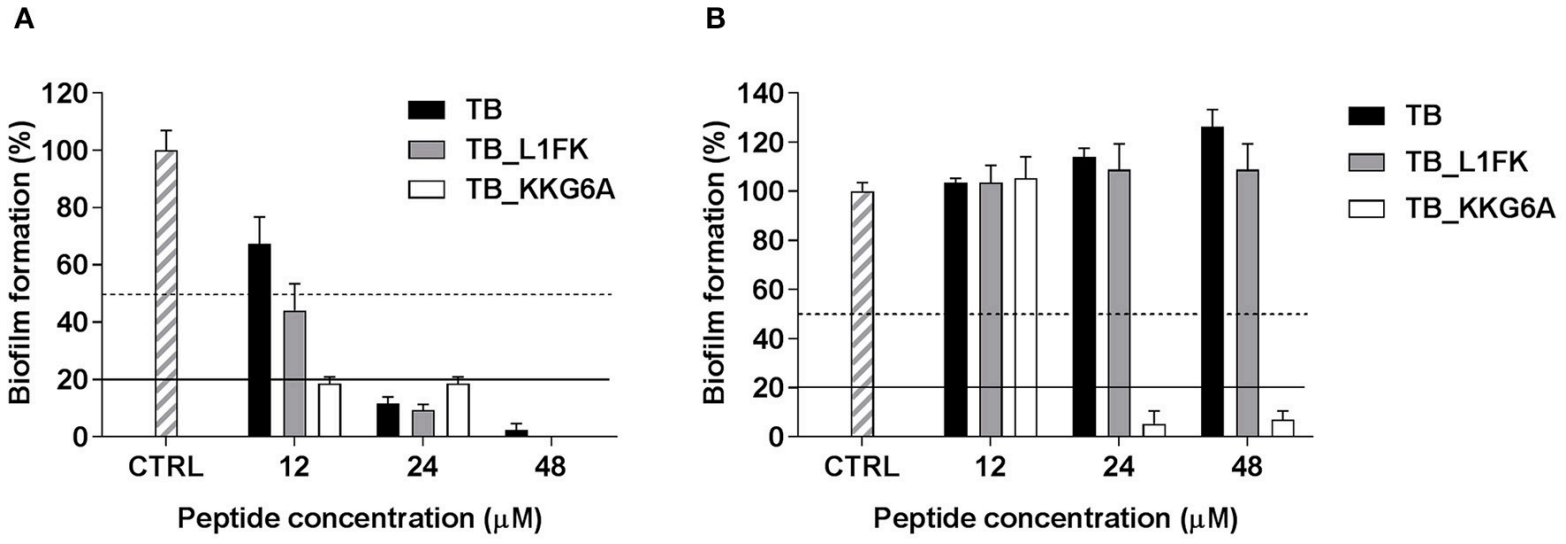
**Inhibitory effect of TB, TB_L1FK, and TB_KKG6A on biofilm formation of *S. aureus* ATCC 33591 (A)** and *P. aeruginosa* ATCC 27853 **(B)**. The inhibitory effect was assessed by measuring the total biofilm biomass by crystal violet staining after 24 h of incubation with the peptides. Control (CTRL) represents untreated bacteria. Dashed and solid lines represent 50 and 80% reduction in biofilm biomass as compared to untreated controls, respectively. Data are reported as mean ± standard error of at least three independent experiments.

We first investigated the ability of TB, TB_L1FK and TB_KKG6A to inhibit the formation of biofilms of *S. aureus* and *P. aeruginosa*, two bacterial species often involved in the formation of biofilms particularly refractory to antimicrobial treatment. The inhibitory effect was assessed by CV staining (total biofilm biomass) evaluating the percentage of biofilm formation after 24 h of incubation with TB or the two TB analogs, as compared to the control biofilms (cells incubated in medium only). As shown in Figure 3A, differently from the parental peptide, TB_L1FK and TB_KKG6A reduced the ability of *S. aureus* to form biofilm of more than 50% as compared to the untreated control at 12 μM. All the peptides caused around 80% decrease of the biofilm biomass at the concentration of 24 μM. When the peptides were assayed against forming biofilms of *P. aeruginosa*, no inhibitory activity of TB and TB_L1FK was observed at concentrations up to 48 μM (Figure 3B). In contrast, TB_KKG6A displayed a considerable ability in reducing the biomass of *P. aeruginosa* biofilms, causing an 80% inhibition at the concentration of 24 μM (Figure 3B).

**Figure 4:**
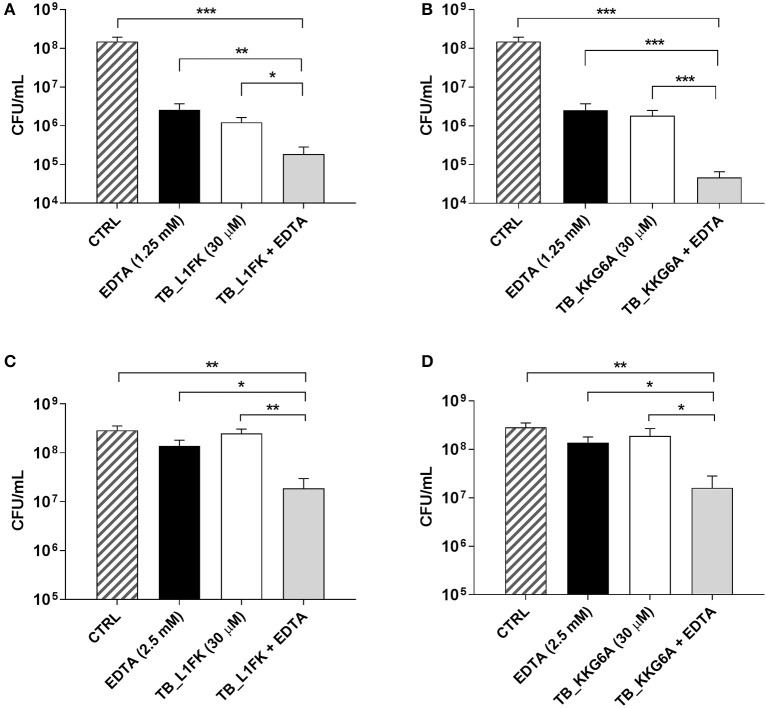
**Activity of TB_L1FK and TB_KKG6A, used alone and in combination with EDTA, against preformed (24-h old) biofilms of *S. aureus* ATCC 33591 (A,B)** and *P. aeruginosa* ATCC 27853 **(C,D)**. The antibiofilm activity of the peptides, EDTA and the peptide-EDTA combinations was evaluated by CFU counting after 24 h of incubation. Control (CTRL) represents untreated biofilms. Data are reported as mean ± standard error of at least three independent experiments. ^*^p < 0.05; ^**^p < 0.01; ^***^p < 0.001 (one way ANOVA followed by Tukey-Kramer *post-hoc* test).

Secondly, the efficacy of TB and its analogs against preformed (24 h-old) biofilms of *S. aureus* and *P. aeruginosa* was evaluated by CFU counting after 24 h of incubation with the peptides. In the case of *S. aureus* biofilms, TB did not exert a considerable antibiofilm activity at concentrations up to 120 μM (data not shown), while TB_L1FK and TB_KKG6A caused a decrease of approximately 2 Log_10_ in the number of biofilm-associated viable cells as compared to untreated biofilms at 30 μM (Figures 4A,B). When tested against biofilms of *P. aeruginosa*, none of the three peptides displayed a significant ability to reduce the number of CFU at the highest tested concentration (120 μM) (data not shown).

### Effect of TB analogs, alone and in combination with EDTA, on preformed biofilms

The possibility to improve the activity of TB analogs against preformed biofilms of *S. aureus* and *P. aeruginosa* was investigated combining the peptides with EDTA, a chelating agent previously reported to enhance the antibiofilm properties of TB (Maisetta et al., 2016). Indeed, the ability of EDTA to establish strong complexes with divalent cations essential for matrix stability could produce a matrix-disaggregating effect and promote the accessibility of peptides to biofilm-forming cells. The antibiofilm activity of various peptide-EDTA combinations was evaluated by CFU counting. Among all the tested combinations, the most powerful potentiating effect in terms of viable count reduction was obtained using both peptides at the concentration of 30 μM in combination with 1.25 mM (for *S. aureus*) or 2.5 mM EDTA (for *P. aeruginosa*). As regards *S. aureus* (Figures 4A,B), the combination of both TB_L1FK and TB_KKG6A with EDTA caused a reduction in the CFU number of approximately 1 Log_10_ (90%) compared to the peptides and EDTA used alone, and 3 Log_10_ (99.9%) compared to control biofilms after 24 h of incubation. Also in the case of *P. aeruginosa*, an enhanced ability of TB_L1FK and TB_KKG6A in biofilm reduction was demonstrated when peptides were used in combination with EDTA. Indeed, both peptide-EDTA combinations reduced the CFU number of approximately 1 Log_10_ as compared to the peptide used alone (Figures 4C,D).

### Effect of TB analogs, alone and in combination with EDTA, on planktonic bacteria in biofilm-like conditions

In order to investigate whether the synergism between TB analogs and EDTA was due to a disaggregating effect on biofilm extracellular matrix and/or to a direct effect on bacterial cells, we assessed the activity of the combination on planktonic bacteria in biofilm-like conditions (i.e., stationary phase cells suspended in BPM) in terms of MIC values. As shown in Table 3, when tested alone, TB_L1FK displayed MICs of 15 and 120 μM against *S. aureus* and *P. aeruginosa*, respectively. In the case of TB_KKG6A, the growth-inhibiting effect was recorded at 7.5 μM for *S. aureus* and at 30 μM for *P. aeruginosa*. In order to identify any synergistic interaction, sub-inhibitory concentrations of each peptide and EDTA were combined and the FIC index for the different peptide-EDTA combinations was calculated. Differently to what observed for the biofilm mode of growth, EDTA was not able to potentiate the antibacterial activity of TB_L1FK and TB_KKG6A against planktonic cells of *S. aureus* (FIC index > 0.5, Table 3). Conversely, a synergistic effect between both TB analogs and EDTA was observed against *P. aeruginosa* planktonic cultures (FIC index = 0.25, Table 3). Interestingly, the combination with EDTA produced an 8-fold decrease in the MIC of both peptides against planktonic *P. aeruginosa* grown in biofilm-like conditions, suggesting a direct effect of EDTA in displacing divalent cations that are required for the integrity of the outer membrane of Gram-negative bacteria (Gray and Wilkinson, 1965; Asbell and Eagon, 1966).

**Table 3:** **MICs of TB_L1FK and TB_KKG6A in biofilm-like conditions against *S. aureus* and *P. aeruginosa* and FIC index of the peptide-EDTA combinations**.

	***S. aureus*** **ATCC 33591**		***P. aeruginosa*** **ATCC 27853**	
	**TB_L1FK**	**TB_KKG6A**	**TB_L1FK**	**TB_KKG6A**
MIC^a^	15	7.5	120	30
FIC index	>0.5	>0.5	0.25 (15 μM)^b^	0.25 (3.75 μM)

a *Concentrations are expressed in μM*.

b *Parentheses include the concentration of the peptide resulting in a synergistic effect*.

### Hemolytic activity

**Figure 5:**
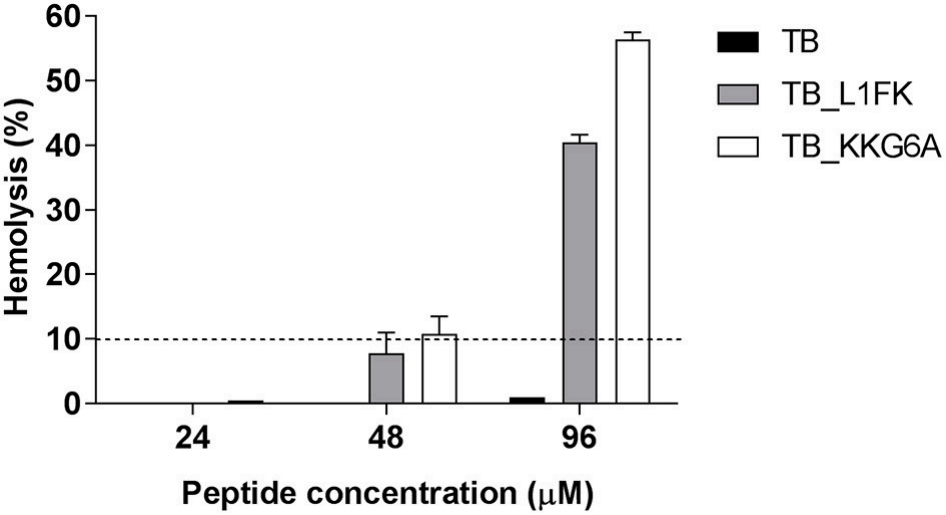
**Hemolytic activity of TB, TB_L1FK, and TB_KKG6A on human erythrocytes after 1 h of incubation at 37°C**. The hemolytic activity was evaluated by the spectrophotometric determination of hemoglobin released from erythrocytes. PBS (0% hemolysis) and Triton X-100 (100% hemolysis) were used as controls. Hemolysis values ≤ 10% (dashed line) are considered to be non-hemolytic (Amin and Dannenfelser, 2006). Data are reported as mean ± standard error of three independent experiments.

The hemolytic activity of TB and TB analogs was evaluated toward human RBCs. As shown in Figure 5, no hemolytic effect of the parental peptide was assessed at concentrations up to 96 μM. An overall increase in hemolytic activity of both analogs was observed. Nevertheless, a hemolysis below 10%, commonly recognized as a safe cut-off (Amin and Dannenfelser, 2006), was observed at concentrations up to 24 μM of TB_KKG6A and up to 48 μM of TB_L1FK.

### Cytotoxicity against PBMCs and A549 cells

**Figure 6:**
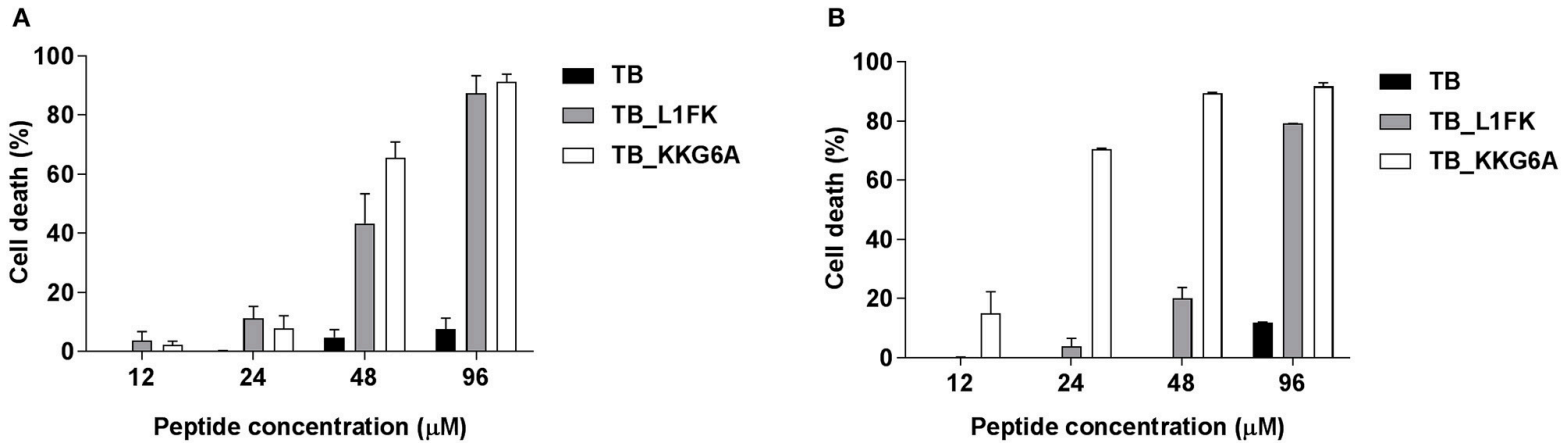
**Cytotoxicity of TB, TB_L1FK, and TB_KKG6A on human PBMCs (A)** and A549 cells **(B)** after 24 h of incubation at 37°C, 5% CO_2_. The cytotoxic activity was evaluated by the PI flow cytometric assay. Cells incubated with culture medium only (100% cell viability) and cells treated with cycloheximide (0% cell viability) were used as controls. Data are reported as mean ± standard error of three independent experiments.

TB, TB_L1FK and TB_KKG6A were tested for cytotoxic activity on PBMCs and A549 cells by flow cytometric determination of PI incorporation in cells treated with different concentrations of the three peptides. As shown in Figure 6, TB did not exhibit a significant cytotoxic effect toward both PBMCs and A549 cells at any of the tested concentrations. Indeed, an approximately 90% viability was observed at 96 μM for both cell types. Both TB analogs displayed higher cytotoxicity against both cell types as compared to TB (Figures 6A,B). When the toxic effect was evaluated as IC_50_ value, TB_L1FK and TB_KKG6A showed comparable levels of cytotoxicity against PBMCs (IC_50_ values of 52 and 49 μM, respectively). In contrast, TB_L1FK displayed lower levels of cytotoxicity against A549 cells with an IC_50_ value of 59 vs. 16 μM of TB_KKG6A.

## Discussion

The use of AMPs as an alternative to conventional antimicrobial agents in the treatment of antibiotic-resistant and/or biofilm-associated infections represents a possibility that is increasingly taken into consideration. Over the last years, a growing body of research has focused on frog skin-derived AMPs with considerable attention being devoted to the antibacterial activity and the mechanism of action of TB (Conlon et al., 2014; Mangoni et al., 2016). It has emerged that such a peptide possesses significant membrane-perturbing properties and folds in a α-helix upon interaction with bacterial membranes (Mangoni et al., 2000). Like most of the members of the temporin family, TB is considerably effective against Gram-positive bacteria, including clinically important multidrug-resistant pathogens, but only poorly active against Gram-negative bacteria (Mangoni et al., 2008). The lower level of activity of TB against these bacteria is likely due to the presence of LPS that induces the oligomerization of the peptide, and hence prevents it to diffuse through the cell wall and reach the target cytoplasmic membrane (Rosenfeld et al., 2006; Mangoni and Shai, 2009). Design of TB analogs with modification of the peptide primary structure may provide peptides with stronger activity against Gram-negative bacterial species and increase the translational potential of TB. Computer-assisted design strategies have led us to obtain TB_L1FK, in which the leucine in position 1 has been replaced by a phenylalanine, the asparagine 7 has been eliminated and an extra lysine has been inserted at the C-terminus increasing the net charge of the peptide. Differently from the traditional optimization procedures, the computational method employed herein allowed to predict the effect of multiple amino acid positions on the antibacterial activity and cytotoxicity of TB, thereby enabling to improve different features of the peptide at the same time and to design a set of candidates for experimental validation. The other analog analyzed in this work, i.e., TB_KKG6A, has been designed by Avitabile and colleagues by replacing the glycine in position 6 with an alanine according to the Ala-scanning method and by adding two lysines at the N-terminus in order to produce a more cationic peptide (Avitabile et al., 2013). Circular dichroism and NMR studies have previously shown that TB_KKG6A strongly interacts with the LPS of the Gram-negative bacterium *E. coli* and assumes a bent helical conformation upon binding (Avitabile et al., 2013; Malgieri et al., 2015).

A comparative analysis of the properties of TB and these two analogs was performed starting from the evaluation of their bactericidal activity against multidrug-resistant bacteria in planktonic form. TB_L1FK and TB_KKG6A displayed an expanded spectrum of action as compared to the parental peptide, being active against all the tested Gram-positive and Gram-negative bacterial strains at very low concentrations. It is likely that the presence of additional positively charged amino acids in their sequence enhanced the affinity of the analogs toward Gram-negative bacteria. This observation is consistent with previous studies, in which optimized analogs of both TB and other temporins (Conlon et al., 2007; Capparelli et al., 2009; Srivastava and Ghosh, 2013) were obtained through the introduction of extra positive charges. Cationic amino acids, such as lysine, play a key role in the interaction of AMPs with the negatively charged components of the bacterial cell surface and the cytoplasmic membrane (Shai, 1999; Hancock and Sahl, 2006). Therefore, an increase in peptide cationicity can promote a more efficient interaction with bacteria, and hence a stronger antibacterial activity (Han et al., 2016). Moreover, faster killing kinetics were observed for the analogs compared to TB against both *S. aureus* and *P. aeruginosa*, selected as representative Gram-positive and Gram-negative bacterial species, respectively. The short time required for peptides to exert their bactericidal effect correlates with the bacterial membrane-permeabilizing activity of the temporin family (Mangoni et al., 2000; Saviello et al., 2010).

The three peptides were also compared regarding their antibiofilm properties using reference strains of *S. aureus* and *P. aeruginosa*. Biofilm-related infections currently represent a relevant clinical problem because of the intrinsic recalcitrance of biofilms to the antibiotic therapy. *S. aureus* and *P. aeruginosa* are common bacterial species involved in biofilm-associated infections, such as wound infections, lung infections in cystic fibrosis patients and implant-related infections (e.g., central venous catheters, endotracheal tubes, prostheses; Ciofu et al., 2015). The ability of these pathogens to produce biofilms is responsible for the establishment of chronic infections, thereby constituting a primary impediment to the complete recovery from infectious diseases (Costerton et al., 1999; Dean et al., 2011). Thus, the identification of new broad-spectrum antibiofilm agents and innovative therapeutic strategies appears as a growing need. To this aim, we explored the efficacy of TB and TB analogs both in preventing biofilm formation and in treating mature biofilms and attempted to enhance the antibiofilm activity of the peptides by combining them with adjuvant compounds. TB analogs showed an improved ability to inhibit the formation of *S. aureus* biofilms at 12 μM, while at 24 μM all three peptides were equally active, causing more than 80% reduction of the biofilm biomass. TB_KKG6A, but not TB_L1FK, showed also a marked activity in inhibiting biofilm formation of *P. aeruginosa* at the concentration of 24 μM. In all cases, the inhibitory activity of the peptides was observed at concentrations close to the MIC values determined in biofilm-like conditions (Table 3), suggesting that the antibiofilm effect was due to the direct killing of biofilm-forming bacteria at their planktonic stage rather than to biofilm-specific mechanisms (Segev-Zarko et al., 2015; Batoni et al., 2016a). When assayed against preformed biofilms, the two analogs, differently from TB, were able to significantly reduce the number of biofilm-associated cells of *S. aureus* at 30 μM, while none of the peptides was effective against *P. aeruginosa* even at 120 μM. It is commonly recognized that preformed biofilms are more challenging to target than the early stages of biofilm formation. The reduced susceptibility of mature biofilms to AMPs is mainly due to the presence of the extracellular matrix that surrounds the bacterial population and constitutes an actual impediment to peptide penetration into the biofilm structure (Otto, 2006; Batoni et al., 2016a). Cationic peptides can be repulsed or sequestrated by the biofilm extracellular polymeric molecules, especially exopolysaccharides and DNA, so that their interaction with bacterial cells can be significantly hampered (Batoni et al., 2016a). In particular, the polysaccharide intracellular adhesin (PIA) of staphylococcal biofilm matrix and alginate, Pel and Psl polysaccharides of *P. aeruginosa* biofilms have been demonstrated to play a major role in the protection from AMPs (Vuong et al., 2004; Chan et al., 2005). Thus, the use of AMPs in combination with compounds able to disaggregate the extracellular matrix could represent a promising strategy to increase their antibiofilm activity and therapeutic potential. In this regard, the chelator EDTA has been shown to reduce the structural integrity of the biofilm of several bacterial species by forming strong complexes with divalent cations (magnesium, calcium, iron) essential for matrix stability (Percival et al., 2005; Banin et al., 2006; Cavaliere et al., 2014; Maisetta et al., 2016). Herein, we combined TB analogs with EDTA in order to improve their efficacy against preformed biofilms of *S. aureus* and *P. aeruginosa*. The combination of TB_L1FK and TB_KKG6A with EDTA resulted in a potentiated antibiofilm effect that led to a statistically significant reduction in the viable count of both bacterial species at a peptide concentration of 30 μM. In order to prove that the enhancement of the antibiofilm activity of TB analogs was actually due to the destabilizing action of EDTA on the biofilm matrix, we also evaluated the effect of the combination peptide-EDTA on planktonic cells in biofilm-like conditions. Interestingly, the peptides exhibited synergy with EDTA against planktonic cultures of *P. aeruginosa*, but not against *S. aureus*. The combination treatment inhibited the growth of *P. aeruginosa* to a greater extent than the peptide used alone, suggesting a direct effect of EDTA also on planktonic bacteria. It is known that divalent cations are key elements in maintaining the integrity of the outer membrane of Gram-negative bacteria as they attenuate the electrostatic repulsive forces between adjacent LPS molecules by forming salt bridges (Gray and Wilkinson, 1965; Asbell and Eagon, 1966). Therefore, chelation of divalent cations by EDTA could enhance the action of the tested AMPs by destabilizing the outer membrane and thus facilitating the peptide access to the bacterial inner membrane. Furthermore, the chelating activity of EDTA may contribute to remove the cationic barrier that prevents the electrostatic interaction of cationic AMPs with the negatively charged bacterial surface (Walkenhorst et al., 2014). Thus, it is likely that EDTA mainly acted as an extracellular matrix-disaggregating agent in the case of *S. aureus* biofilms, facilitating the diffusion of the peptides through the biofilm layers. On the other hand, in the case of *P. aeruginosa* biofilms, the enhanced effect of the peptide-EDTA combinations could be very well due not only to the perturbing effect on the extracellular matrix, but also on a direct effect on biofilm-embedded cells.

The evaluation of the cytotoxicity of AMPs toward the host cells is an essential step to their development as therapeutics. It is generally accepted that there is a direct relationship between the antimicrobial potency of AMPs and their cytotoxic properties (Takahashi et al., 2010). A subtle balance of several physicochemical and structural parameters (cationicity, amphipathicity, hydrophobicity, and helicity) is necessary to ensure the maximum antibacterial efficacy and target cell selectivity of the peptides (Chen et al., 2005; Zelezetsky et al., 2005). Therefore, we evaluated the hemolytic effect of TB analogs on human erythrocytes and their cytotoxic activity on human PBMCs and the human-derived epithelial cell line A549. Along with the enhancement of the antimicrobial activity, modifications in TB sequence led to an overall increase of the hemolytic activity and cytotoxicity of the native peptide. Nevertheless, both TB_L1FK and TB_KKG6A were non-hemolytic at concentrations that resulted to be active against both planktonic and biofilm-growing bacteria. A percentage of hemolysis lower than 10% was assessed at peptide concentrations close to that used in combination with EDTA in treating mature biofilms of *S. aureus* and *P. aeruginosa*. When tested against mammalian cells, TB_L1FK resulted less cytotoxic than TB_KKG6A against human epithelial cells, suggesting that the computational method employed generated a sequence showing a good compromise between antibacterial and cytotoxic activity and promising features for topic applications. In the case of PBMCs, both TB analogs displayed comparable and quite high levels of cytotoxicity. A promising solution to reduce the toxicity of AMPs is the development of appropriate delivery systems for their controlled and/or targeted release. In this regard, our group has recently developed a chitosan-based nanostructured delivery system loaded with TB that ensured a considerable reduction of the cytotoxic activity of the peptide toward mammalian cells (Piras et al., 2015).

## Conclusions

In the present study, we performed a detailed characterization of the bactericidal and antibiofilm activity of TB analogs in order to demonstrate the potential of computational peptide design in the improvement of the antimicrobial properties of AMPs. The introduction of appropriate modifications in the primary sequence of TB led to optimized analogs with a stronger and faster bactericidal activity and a wider spectrum of action as compared to the parental peptide. Furthermore, TB analogs exhibited an improved ability both in preventing biofilm formation and in treating preformed biofilms of *S. aureus* and *P. aeruginosa*, especially when used in combination with EDTA. The antibiofilm action of the peptide-EDTA combination was likely due to a disaggregating effect on the biofilm extracellular matrix and/or to a direct effect on bacterial cells. Collectively, our results suggest that TB analogs represent a promising template for the development of novel antimicrobials for the treatment of antibiotic-resistant and/or biofilm-associated infections. In this regard, current work is devoted to the development of a nanostructured delivery system for TB analogs with the aim to reduce their toxicity and to control their pharmacokinetics, thus further improving the therapeutic potential of these molecules.

## Author contributions

LG, GAM, SE, and GB: conception and design of the work; acquisition, analysis, and interpretation of the data for the work; GM: design and analysis of TB_L1FK; LG, GAM, GM, and GB: drafting of the work; LG, GAM, GM, SE, and GB: critical revision of the work; final approval.

## Funding

This work was supported by funds from University of Pisa (Rating di Ateneo).

### Conflict of interest statement

The authors declare that the research was conducted in the absence of any commercial or financial relationships that could be construed as a potential conflict of interest.

## Abbreviations

AMP: antimicrobial peptide
BPM: biofilm promoting medium
BSA: bovine serum albumin
CCS: combined consensus scale
MCC: Mathews correlation coefficient
MOEA: multi-objective evolutional algorithms
CFU: colony-forming units
CV: crystal violet
EDTA: ethylenediaminetetraacetic acid
EPS: extracellular polymeric substance
FCS: fetal calf serum
FIC: fractional inhibitory concentration
LPS: lipopolysaccharide
MBC: minimal bactericidal concentration
MIC: minimal inhibitory concentration
PBMCs: peripheral blood mononuclear cells
PBS: phosphate buffered saline
PI: propidium iodide
RBCs: red blood cells
SPB: sodium-phosphate buffer
TB: temporin 1Tb
TSA: tryptone soy agar
TSB: tryptone soy broth.
