# Supplementary material for: Analogs of the Frog-skin Antimicrobial Peptide Temporin 1Tb Exhibit a Wider Spectrum of Activity and a Stronger Antibiofilm Potential as Compared to the Parental Peptide
Source: Front Chem. 2017 Apr 11;5:24. doi: 10.3389/fchem.2017.00024 (PMC5387044; doi:10.3389/fchem.2017.00024)
Supplement: Supplementary file 1 [file DataSheet1.PDF]

Supplementary Material:

Analogues of the Frog-skin Antimicrobial Peptide Temporin 1Tb Exhibit a Wider Spectrum of Activity and a Stronger Antibiofilm Potential as Compared to the Parental Peptide

Lucia Grassi, Giuseppantonio Maisetta, Giuseppe Maccari, Semih Esin, Giovanna Batoni *

***Correspondence:**
Giovanna Batoni
giovanna.batoni@med.unipi.it

1 SUPPLEMENTARY DATA

1.1 Dataset preparation

A dataset representing peptides with cytotoxic activity was designed with the aim to train and validate a statistical model able to discern between ‘toxic’ and ‘non-toxic’ peptides, giving a confidence score. A set of sequences ranging from 9 to 35 amino acids length was collected from different bioactive peptide databases, as previously described (Gupta et al, 2013). After removal of peptides with non-standard residues, 1709 peptides were left. The negative dataset was populated with non-secretory sequences randomly extracted from UniProt database, without the ‘antimicrobic’ and ‘cytotoxic’ annotation and with a length ranging from 9 to 35 amino acids, for a total count of 2010 negative sequences. A homology cut-off was imposed to exclude similar peptides in order to avoid redundant data that could influence the prediction performance. Peptides showing a sequence identity equal or greater than 70% to any other in the dataset were identified and removed by the CD-HIT (Cluster Database at High Identity with Tolerance) program (Li and Godzik, 2006).

1.2 Data encoding

In order to build a statistical model, able to discern between toxic and non-toxic peptides, each sequence in the dataset was encoded into computer-intelligible variables representing peptides physicochemical peculiarities. Peptide charge at different pH conditions, isoelectric point and molecular weight, together with the z-scale moment, were used to describe global features of the peptide sequences. Z-scale descriptors (Hellberg et al, 1987) are highly condensed variables, originally derived from a principal component analysis (PCA) of several experimental and theoretical physicochemical properties for the 20 naturally occurring amino acids (AAs). These descriptors were successively expanded to include artificial AAs for a total of 87 AAs (Sandberg et al, 1998). In detail, this latter version corresponds to the first five principal components explaining the variance in the set: z_1, z_2, and z_3 represent the AA hydrophobicity, steric properties, and polarity, respectively, while z_4 and z_5 describe the electronic effects of the residues. The z-scale moment (μZ_i), an extension of Eisenberg’s hydrophobic moment equation (Eisenberg at al, 1982), represents z-scales distribution along peptide sequences.

$$\mu Z_i = \sqrt{\left(\sum_{k=1}^{L} Z_i^k \sin(\delta k)\right)^2 + \left(\sum_{k=1}^{L} Z_i^k \cos(\delta k)\right)^2}$$

Equation 1. Z-scale moment

In Equation 1, δ is the angular frequency of the AA residues forming the structure (100° for alpha helix); k is the number of the particular residue examined, L is the length of the sequence and Z_i^k is the z_i-scale value of the kth AA. In particular, μZ_1 represents a measure of the hydrophobicity distribution along peptide sequence. Topological descriptors represent the interaction of different residues along the amino acidic sequence and are used to keep into account peptide's secondary structure. QSAR descriptors were encoded into auto- and cross covariance (ACC) values. Classical ACC transformation was introduced by Wold et al. (Wold et al, 1993) and results in two kinds of variables: auto covariance (AC) of the same descriptor and cross covariance (CC) between two different descriptors. Briefly, for a given protein sequence, ACC variables describe the average interactions between residues distributed a certain *lag* apart throughout the whole sequence. In this work, the *Minimum and Maximum of auto- and cross-covariances* (mMACC) algorithm is used (Maccari et al, 2013), weak and strong correlations are kept into account (Equation 2).

$$AC_{\min d} = MIN\left[Z_i^k * Z_i^{k+d}\right]; AC_{\max d} = MAX\left[Z_i^k * Z_i^{k+d}\right](k = 1,2,3..L-d)$$
$$CC_{\min d} = MIN\left[Z_i^k * Z_i^{k+d}\right]; CC_{\max d} = MAX\left[Z_i^k * Z_i^{k+d}\right](k = 1,2,3..L-d)$$

Equation 2. Minimum and Maximum of auto and cross-covariance equations

Both in the global and topological descriptors, Z-scale values were mean-centered and scaled prior to their use, as described by the following equation:

$$Z_i = \frac{z_i - \frac{1}{N}\sum_{k=1}^{N} z_i^k}{\sqrt{\frac{1}{N}\sum_{j=1}^{N}\left[z_i^j - \frac{1}{N}\sum_{k=1}^{N} z_i^k\right]^2}}$$

Equation 3. Z-scale descriptor normalization

Where Z_i is the ith descriptor of z-scales variables, z_i is the original z-scale value and N is the number of AAs in the z-scales descriptors table.

1.3 Feature selection and model generation

In this study, the Random Forest algorithm (RF), implemented in the software suite WEKA (Witten et al, 2011), was adopted as prediction engine. Model performance was measured with a 10-fold cross-validation analysis, where each dataset was divided into 10 parts - 9 parts for model learning (training) and the remaining part for validation (testing). As a performance measure, the Matthews correlation coefficient (MCC) was used, as defined below.

$$\text{Sensitivity} = \frac{TP}{TP+FN}$$

$$\text{Precision} = \frac{TP}{TP+FP}$$

$$\text{Accuracy} = \frac{TP+TN}{TP+TN+FP+FN}$$

$$MCC = \frac{(TP*TN)-(FN*FP)}{\sqrt{(TP+FN)*(TN+FP)*(TP+FP)*(TN+FN)}}$$

Equation 4. Performance evaluation equations

Where *TP*, *TN*, *FP* and *FN* are the number of true positive, true negative, false positive and false negative, respectively, resulting from the model. MCC is an important index used to evaluate the performance of the predictor when the dataset is not balanced (Baldi et al, 2000). In order to obtain a non-redundant set of descriptors, the Maximum Relevance, Minimum Redundancy (mRMR) method (Peng et al, 2005) was employed to sort features in descending order of importance. Incremental Feature Selection (IFS) (Huang et al, 2010) was applied to the sorted descriptors list by consecutively incrementing by 5 the number of descriptors. Each descriptor set thus obtained was evaluated by tenfold cross-validation and the IFS curve was plotted to unveil the relation between the performance of the model and the feature subset. The optimal feature subset is defined as that showing the highest MCC value (**Figure S1**); the selected model was used for peptides classification. A description of the applied descriptors is available in **Table S1**, while the hierarchical list of the final descriptors is shown in **Table S2**.

1.4 Sequence similarity

For TB peptide optimization, a supplemental objective representing sequence similarity was added. Sequence similarity is defined by the Smith-Waterman score between the respective peptide sequences (Smith at al, 1981). However, since the Smith-Waterman score is dependent on input sequences length, the final score was normalized between 0 and 1 by dividing by the maximum score of the two self-alignments, as shown in Equation 5 (Zang et al, 2012).

$$NS_{A,B} = \frac{S_{A,B}}{\max\left(S_{A,A}, S_{B,B}\right)}$$

Equation 5. Smith-Waterman normalized score

Here, $S_{A,B}$ is the similarity score between sequence A and B, $S_{A,A}$ and $S_{B,B}$ are the self-alignment score of sequence A and sequence B, respectively. In order to consider not only the identity between two amino acidic positions, a score matrix was defined by calculating the Euclidean distance between the five auto-scaled z-scale values of each AA pairs.

References

Baldi P., Brunak S., Chauvin Y., Andersen C.A.F., Nielsen H. (2000). Assessing the accuracy of prediction algorithms for classification: an overview. *Bioinformatics.* 16: 412-424.

Eisenberg D., Weiss R.M.,. Terwilliger T.C. (1982). The helical hydrophobic moment: a measure of the amphiphilicity of a helix. *Nature* 299: 371-374.

Gupta, S., Kapoor, P., Chaudhary, K., Gautam, A., Kumar, R., Raghava, G.P.S. (2013). In silico approach for predicting toxicity of peptides and proteins. *PLoS One.* 8:e73957. doi: 10.1371/journal.pone.0073957.

Hellberg S., Sjöström M., Skagerberg B., Wold S. (1987). Peptide quantitative structure activity relationship, a multivariate approach. *J. Med. Chem*. 30: 1126-1135.

Huang T., Shi X.H., Wang P., He Z., Feng K.Y., Hu L., Kong X., Li Y.X., Cai Y.D., Chou K.C. (2010). Analysis and prediction of the metabolic stability of proteins based on their sequential features, subcellular locations and interaction networks. *PLoS One.* 5: e10972.

Li, W., Godzik, A. (2006). Cd-hit: a fast program for clustering and comparing large sets of protein or nucleotide sequences. *Bioinformatics.* 22: 1658-1659. doi: 10.1093/bioinformatics/btl158.

Maccari G., Di Luca M., Nifosí R., Cardarelli F., Signore G., Boccardi C., Bifone A. (2013). Antimicrobial peptides design by evolutionary multiobjective optimization. *PLoS Comput Biol.* 9: e1003212.

Peng H., Long F., Ding C. (2005). Feature selection based on mutual information: criteria of max-dependency, max-relevance, and min-redundancy. *IEEE Trans. Pattern Anal. Mach. Intell.* 27: 1226-1238.

Sandberg M., Eriksson L., Jonsson J., Sjöström M., Wold S. (1998). New chemical descriptors relevant for the design of biologically active peptides. A multivariate characterization of 87 amino acids. *J. Med. Chem*. 41: 2481-2491.

Smith T.F., Waterman M.S. (1981). Identification of common molecular subsequences. *J. Mol. Biol.* 147: 195–197.

Witten I.H., Frank E., Hall M.A. (2011). Data mining: practical machine learning tools and techniques. Morgan Kaufmann, Burlington, MA

Wold S., Jonsson J., Sjöström M., Sandberg M., Rännar S. (1993). DNA and peptide sequences and chemical processes multivariately modelled by principal component analysis and partial least-squares projections to latent structures. *Ann. Chim. Acta*. 277: 239-253.

Zang M., Leong H. (2012). BBH-LS: an algorithm for computing positional homologs using sequence and gene context similarity. *BMC Syst. Biol.* 6: S22.

2 SUPPLEMENTARY TABLES AND FIGURES

2.1 Tables

Table S1. Applied descriptors in the model building. A list of the applied descriptors with abbreviation and description is provided.

Type	Abbreviation	Description
**Global**	NetCharge@5	Net charge at pH = 5.
	NetCharge@7	Net charge at pH = 7.
	NetCharge@9	Net charge at pH = 9.
	Wimley White (pH n)	Wimley White partitioning at pH *n*
	Isoelectric point	Peptide's isoelectric point
	Size	Total amino acid count.
	Property_Zn	Z-scale average sum of property *n* along peptide sequence.
	Variable Moment Z*n* (100 D)	Z-scale moment distribution of property n along peptide sequence at 100 degrees (the angle between two residues in alpha helix conformation)
**Topological**	D_X_AC_LAG_N_[MIN,MAX];<br>D_X:Y_CC_LAG_N_[MIN,MAX]	D_X_AC_LAG_N_[MIN,MAX]: Topological descriptor of the auto covariance of descriptor X with a lag of N.<br>D_X:Y_CC_LAG_N_[MIN,MAX]: Topological descriptor of the cross covariance between descriptor X and Y, with a lag of N.<br>With X and Y being a value between 0 and 4:<br>0) Z-scale Descriptor 1<br>1) Z-scale Descriptor 2<br>2) Z-scale Descriptor 3<br>3) Z-scale Descriptor 4<br>4) Z-scale Descriptor 5

Table S2. Hierarchical list of descriptors. List of descriptors sorted by the mRMR method.

	Name	#	Name	#	Name	#	Name	#	Name	#	Name
2	Property_z5_10	117	D_4:0_CC_LAG_3_MIN	232	D_0:2_CC_LAG_5_MIN	347	D_1:3_CC_LAG_5_MIN	462	D_1:0_CC_LAG_7_MAX	577	D_4:3_CC_LAG_1_MIN
3	Property_z2_6	118	D_0:1_CC_LAG_4_MIN	233	D_0:4_CC_LAG_5_MIN	348	D_1_AC_LAG_4_MIN	463	D_2:1_CC_LAG_3_MAX	578	D_3:1_CC_LAG_9_MAX
4	Property_z3_14	119	Property_z2_12	234	D_0:4_CC_LAG_8_MAX	349	D_2:0_CC_LAG_7_MIN	464	D_0_AC_LAG_8_MIN	579	Property_z3_20
5	Property_z2_2	120	Property_z3_16	235	D_1:0_CC_LAG_4_MIN	350	D_0_AC_LAG_6_MIN	465	D_3:1_CC_LAG_1_MAX	580	D_2:3_CC_LAG_0_MAX
6	Property_z5_9	121	D_4_AC_LAG_0_MIN	236	D_2:1_CC_LAG_3_MIN	351	D_2:1_CC_LAG_6_MAX	466	D_4:3_CC_LAG_8_MIN	581	D_2:3_CC_LAG_5_MIN
7	Property_z2_3	122	D_0:2_CC_LAG_0_MIN	237	D_4_AC_LAG_6_MAX	352	D_0:2_CC_LAG_8_MIN	467	Property_z1_31	582	Property_z1_29
8	Property_z2_1	123	D_1:0_CC_LAG_1_MIN	238	D_3:0_CC_LAG_3_MAX	353	D_4:0_CC_LAG_9_MIN	468	D_3_AC_LAG_4_MAX	583	D_1:2_CC_LAG_2_MAX
9	Property_z5_6	124	D_4:1_CC_LAG_6_MAX	239	D_0:3_CC_LAG_4_MIN	354	D_1:0_CC_LAG_2_MAX	469	D_4:2_CC_LAG_9_MAX	584	D_2:1_CC_LAG_9_MAX
10	Property_z4_5	125	Property_z4_34	240	D_1:4_CC_LAG_3_MIN	355	D_2:0_CC_LAG_3_MAX	470	D_1_AC_LAG_5_MAX	585	D_4:3_CC_LAG_4_MIN
11	Property_z2_34	126	D_0:4_CC_LAG_1_MAX	241	D_1:3_CC_LAG_3_MAX	356	D_3:1_CC_LAG_9_MIN	471	D_3:2_CC_LAG_5_MIN	586	D_2:4_CC_LAG_8_MIN
12	Property_z2_9	127	D_2:1_CC_LAG_2_MIN	242	D_4_AC_LAG_4_MAX	357	D_3_AC_LAG_0_MIN	472	Property_z2_16	587	Property_z2_18
13	Property_z2_7	128	D_0:4_CC_LAG_1_MIN	243	Property_z5_14	358	D_0_AC_LAG_7_MAX	473	D_2:1_CC_LAG_9_MIN	588	D_4:2_CC_LAG_0_MIN
14	Property_z1_4	129	D_3:0_CC_LAG_1_MAX	244	D_0:2_CC_LAG_8_MAX	359	D_1:4_CC_LAG_5_MIN	474	D_1_AC_LAG_6_MAX	589	D_3:2_CC_LAG_3_MAX
15	Property_z2_11	130	Property_z5_12	245	Property_z5_33	360	Property_z5_32	475	D_3:4_CC_LAG_2_MIN	590	Property_z2_29
16	Property_z3_1	131	D_4:0_CC_LAG_7_MAX	246	D_0:1_CC_LAG_3_MAX	361	D_2:0_CC_LAG_6_MIN	476	D_3_AC_LAG_8_MAX	591	Property_z3_22
17	D_1:2_CC_LAG_3_MIN	132	Property_z4_0	247	D_0:1_CC_LAG_7_MIN	362	D_1:2_CC_LAG_0_MAX	477	D_1:2_CC_LAG_8_MAX	592	D_0:2_CC_LAG_7_MAX
18	Property_z1_9	133	D_0:1_CC_LAG_0_MIN	248	D_0_AC_LAG_1_MIN	363	D_3:2_CC_LAG_0_MIN	478	Property_z2_31	593	D_4:2_CC_LAG_5_MIN
19	Property_z3_3	134	D_4:1_CC_LAG_1_MAX	249	D_4:3_CC_LAG_7_MAX	364	D_1:4_CC_LAG_9_MAX	479	D_1:3_CC_LAG_8_MAX	594	D_2:4_CC_LAG_5_MIN
20	Property_z2_10	135	D_4:1_CC_LAG_1_MIN	250	D_3:1_CC_LAG_2_MIN	365	D_1:3_CC_LAG_3_MIN	480	D_2:1_CC_LAG_8_MAX	595	Property_z3_21
21	Property_z1_2	136	Property_z2_13	251	D_0:2_CC_LAG_4_MIN	366	D_0:3_CC_LAG_8_MIN	481	D_2:3_CC_LAG_9_MIN	596	D_3:4_CC_LAG_5_MIN
22	Property_z2_8	137	D_3:0_CC_LAG_4_MIN	252	D_3_AC_LAG_7_MIN	367	D_0:1_CC_LAG_8_MAX	482	D_4:2_CC_LAG_5_MAX	597	D_2:0_CC_LAG_0_MAX

23	Property_z4_4	138	D_0:4_CC_LAG_7_MAX	253	D_4:0_CC_LAG_4_MIN	368	D_0_AC_LAG_5_MIN	483	Wimley-White Partitioning (pH9.0)	598	D_2:4_CC_LAG_2_MIN
24	Property_z3_12	139	D_3:4_CC_LAG_0_MAX	254	D_1:4_CC_LAG_7_MAX	369	D_4:3_CC_LAG_6_MIN	484	D_3:2_CC_LAG_7_MIN	599	Property_z3_26
25	Variable moment (z2:100.0D)	140	D_4_AC_LAG_1_MAX	255	D_0:3_CC_LAG_4_MAX	370	D_3:1_CC_LAG_7_MIN	485	Property_z3_28	600	D_0:2_CC_LAG_6_MAX
26	Property_z2_0	141	D_0_AC_LAG_3_MIN	256	D_0:1_CC_LAG_1_MIN	371	D_3_AC_LAG_7_MAX	486	D_3_AC_LAG_3_MAX	601	D_3:2_CC_LAG_0_MAX
27	Property_z1_8	142	D_1:3_CC_LAG_8_MIN	257	D_1:4_CC_LAG_6_MIN	372	D_1_AC_LAG_3_MIN	487	D_1:0_CC_LAG_6_MAX	602	Property_z4_16
28	Property_z3_7	143	D_4:0_CC_LAG_1_MIN	258	D_4_AC_LAG_0_MAX	373	D_1:2_CC_LAG_5_MAX	488	D_2:0_CC_LAG_7_MAX	603	D_4:3_CC_LAG_3_MIN
29	D_4_AC_LAG_5_MIN	144	Property_z3_34	259	D_0:1_CC_LAG_2_MAX	374	NetCharge@5.0	489	Property_z5_16	604	Property_z3_25
30	Property_z1_5	145	D_2:1_CC_LAG_4_MIN	260	D_0:2_CC_LAG_6_MIN	375	D_4:1_CC_LAG_9_MAX	490	D_2:3_CC_LAG_3_MIN	605	D_2:4_CC_LAG_7_MIN
31	Property_z3_2	146	D_4:3_CC_LAG_3_MAX	261	D_4:3_CC_LAG_5_MIN	376	Property_z4_32	491	D_3:0_CC_LAG_8_MAX	606	D_3:2_CC_LAG_5_MAX
32	Property_z1_11	147	D_4:3_CC_LAG_0_MAX	262	D_4:0_CC_LAG_8_MIN	377	D_3:0_CC_LAG_5_MAX	492	D_3:4_CC_LAG_8_MIN	607	D_2:3_CC_LAG_9_MAX
33	Property_z5_7	148	D_3:0_CC_LAG_5_MIN	263	D_0_AC_LAG_4_MAX	378	D_1:3_CC_LAG_6_MIN	493	D_1_AC_LAG_9_MIN	608	D_2:3_CC_LAG_4_MAX
34	Property_z3_0	149	D_4:0_CC_LAG_4_MAX	264	D_2:0_CC_LAG_2_MIN	379	D_0:1_CC_LAG_9_MIN	494	D_3:1_CC_LAG_6_MAX	609	Property_z2_19
35	Property_z5_1	150	Property_z1_17	265	D_3:1_CC_LAG_8_MIN	380	D_4:2_CC_LAG_0_MAX	495	Property_z4_30	610	D_2:4_CC_LAG_1_MIN
36	Property_z3_9	151	D_0:2_CC_LAG_3_MIN	266	Property_z4_33	381	D_0_AC_LAG_4_MIN	496	D_4:3_CC_LAG_7_MIN	611	D_3:4_CC_LAG_3_MIN
37	Property_z5_11	152	D_1_AC_LAG_0_MAX	267	D_3:4_CC_LAG_2_MAX	382	D_1:3_CC_LAG_4_MAX	497	D_3:2_CC_LAG_6_MIN	612	Property_z1_28
38	D_4:1_CC_LAG_0_MAX	153	D_4_AC_LAG_2_MAX	268	D_1:2_CC_LAG_7_MIN	383	D_3:0_CC_LAG_6_MAX	498	D_2:1_CC_LAG_4_MAX	613	D_3:2_CC_LAG_6_MAX
39	Property_z5_2	154	D_1:0_CC_LAG_3_MIN	269	D_0:1_CC_LAG_0_MAX	384	D_4:2_CC_LAG_8_MIN	499	Property_z1_22	614	D_4:2_CC_LAG_6_MIN
40	Variable moment (z3:100.0D)	155	D_1:4_CC_LAG_1_MIN	270	D_4:1_CC_LAG_2_MIN	385	Property_z5_15	500	D_1_AC_LAG_8_MAX	615	Property_z1_23
41	D_4:0_CC_LAG_1_MAX	156	Property_z2_14	271	D_2:4_CC_LAG_4_MAX	386	D_1_AC_LAG_1_MAX	501	D_1:2_CC_LAG_1_MAX	616	D_3:2_CC_LAG_8_MAX
42	Property_z5_8	157	D_0:3_CC_LAG_1_MAX	272	D_4_AC_LAG_1_MIN	387	D_2:1_CC_LAG_7_MAX	502	D_3_AC_LAG_4_MIN	617	D_2:4_CC_LAG_0_MIN
43	Property_z1_13	158	D_4:0_CC_LAG_0_MAX	273	D_3:4_CC_LAG_7_MAX	388	D_2:0_CC_LAG_8_MIN	503	Property_z5_18	618	D_3:2_CC_LAG_7_MAX
44	D_0:4_CC_LAG_3_MIN	159	Property_z5_34	274	D_0:1_CC_LAG_5_MAX	389	D_0_AC_LAG_1_MAX	504	D_2:3_CC_LAG_6_MIN	619	Property_z5_28
45	D_2:0_CC_LAG_0_MIN	160	D_0:3_CC_LAG_2_MIN	275	D_0:3_CC_LAG_3_MIN	390	D_1:4_CC_LAG_4_MIN	505	D_4:2_CC_LAG_3_MAX	620	D_2:0_CC_LAG_2_MAX
46	Property_z4_12	161	D_4:0_CC_LAG_5_MAX	276	NetCharge@7.0	391	D_1:3_CC_LAG_7_MAX	506	D_1_AC_LAG_5_MIN	621	D_2:4_CC_LAG_4_MIN

47	Property_z1_1	162	D_2:0_CC_LAG_1_MIN	277	D_1:3_CC_LAG_7_MIN	392	D_3:1_CC_LAG_3_MIN	507	Property_z5_30	622	Property_z4_17
48	Property_z3_8	163	D_1:0_CC_LAG_8_MIN	278	D_4_AC_LAG_8_MIN	393	D_1:3_CC_LAG_5_MAX	508	Property_z1_20	623	Property_z2_24
49	D_3:1_CC_LAG_4_MIN	164	D_4_AC_LAG_8_MAX	279	D_1:0_CC_LAG_0_MAX	394	D_1:2_CC_LAG_9_MIN	509	D_3:2_CC_LAG_8_MIN	624	D_3:4_CC_LAG_4_MIN
50	D_4:3_CC_LAG_4_MAX	165	D_3:1_CC_LAG_1_MIN	280	D_0:4_CC_LAG_6_MIN	395	D_0:3_CC_LAG_8_MAX	510	D_2:3_CC_LAG_8_MAX	625	D_0:2_CC_LAG_1_MAX
51	D_3:0_CC_LAG_0_MIN	166	D_1_AC_LAG_2_MIN	281	D_4_AC_LAG_2_MIN	396	D_4:2_CC_LAG_6_MAX	511	D_1_AC_LAG_6_MIN	626	Property_z2_28
52	Variable moment (z1:100.0D)	167	D_4:0_CC_LAG_6_MIN	282	D_2:1_CC_LAG_6_MIN	397	D_3:1_CC_LAG_5_MAX	512	D_2:4_CC_LAG_6_MAX	627	D_4:2_CC_LAG_8_MAX
53	Property_z3_10	168	D_4:3_CC_LAG_2_MAX	283	D_1_AC_LAG_1_MIN	398	D_0:2_CC_LAG_9_MIN	513	D_3_AC_LAG_3_MIN	628	D_2:4_CC_LAG_3_MIN
54	Property_z4_6	169	D_0:4_CC_LAG_3_MAX	284	D_0_AC_LAG_3_MAX	399	D_3:0_CC_LAG_7_MAX	514	Property_z1_19	629	D_2:0_CC_LAG_1_MAX
55	Property_z2_5	170	D_0:2_CC_LAG_2_MIN	285	D_3:0_CC_LAG_8_MIN	400	D_4:1_CC_LAG_8_MIN	515	D_0:2_CC_LAG_0_MAX	630	Property_z2_21
56	Property_z4_11	171	D_0:1_CC_LAG_5_MIN	286	Property_z1_32	401	D_3:1_CC_LAG_2_MAX	516	D_2:3_CC_LAG_0_MIN	631	D_3:4_CC_LAG_0_MIN
57	Property_z1_3	172	D_4:0_CC_LAG_2_MIN	287	D_2:4_CC_LAG_1_MAX	402	D_2:4_CC_LAG_9_MAX	517	D_1:3_CC_LAG_9_MAX	632	Property_z4_28
58	Variable moment (z4:100.0D)	173	D_3:0_CC_LAG_0_MAX	288	D_2:1_CC_LAG_0_MIN	403	D_0_AC_LAG_8_MAX	518	Property_z2_30	633	D_2:3_CC_LAG_2_MAX
59	D_0:4_CC_LAG_2_MAX	174	D_0:4_CC_LAG_4_MAX	289	D_1:3_CC_LAG_2_MAX	404	D_0:4_CC_LAG_9_MIN	519	D_3_AC_LAG_8_MIN	634	Property_z2_23
60	Property_z2_4	175	D_1:0_CC_LAG_2_MIN	290	D_0:4_CC_LAG_9_MAX	405	D_1:0_CC_LAG_4_MAX	520	D_3:4_CC_LAG_9_MIN	635	D_3:2_CC_LAG_9_MAX
61	Property_z4_7	176	D_1:4_CC_LAG_2_MAX	291	D_4:0_CC_LAG_7_MIN	406	D_1:3_CC_LAG_0_MAX	521	D_4:2_CC_LAG_9_MIN	636	D_0:2_CC_LAG_5_MAX
62	D_1:2_CC_LAG_0_MIN	177	D_4:3_CC_LAG_2_MIN	292	D_4:1_CC_LAG_7_MIN	407	D_1:4_CC_LAG_8_MIN	522	Property_z3_18	637	D_2:3_CC_LAG_1_MAX
63	D_1:0_CC_LAG_5_MIN	178	D_4_AC_LAG_7_MIN	293	D_3:4_CC_LAG_5_MAX	408	D_3:2_CC_LAG_4_MIN	523	D_2:4_CC_LAG_2_MAX	638	Property_z2_22
64	Property_z1_12	179	D_0_AC_LAG_2_MIN	294	D_1:2_CC_LAG_8_MIN	409	D_0:3_CC_LAG_9_MIN	524	D_4:2_CC_LAG_1_MIN	639	D_3:4_CC_LAG_1_MIN
65	Property_z4_2	180	D_2:1_CC_LAG_7_MIN	295	D_4_AC_LAG_7_MAX	410	D_2:4_CC_LAG_0_MAX	525	D_2:0_CC_LAG_9_MAX	640	Property_z3_24
66	D_1:4_CC_LAG_1_MAX	181	D_4:1_CC_LAG_5_MAX	296	D_4:1_CC_LAG_8_MAX	411	D_1_AC_LAG_7_MIN	526	Property_z3_23	641	D_2:3_CC_LAG_3_MAX
67	Property_z3_13	182	D_3:0_CC_LAG_6_MIN	297	D_0:4_CC_LAG_7_MIN	412	Property_z1_18	527	D_1_AC_LAG_9_MAX	642	Property_z4_27
68	Property_z4_9	183	D_1:4_CC_LAG_0_MIN	298	Wimley-White Partitioning (pH5.0)	413	D_3:1_CC_LAG_0_MAX	528	D_3:2_CC_LAG_4_MAX	643	D_2_AC_LAG_9_MIN
69	Variable moment (z5:100.0D)	184	D_3:4_CC_LAG_1_MAX	299	D_4_AC_LAG_3_MIN	414	D_2:3_CC_LAG_8_MIN	529	D_2:3_CC_LAG_7_MIN	644	Property_z2_20
70	Property_z4_1	185	D_1:2_CC_LAG_6_MIN	300	D_1:3_CC_LAG_1_MIN	415	D_0:3_CC_LAG_9_MAX	530	D_3_AC_LAG_2_MAX	645	Property_z5_27

71	Property_z4_10	186	D_4_AC_LAG_3_MAX	301	D_1:4_CC_LAG_8_MAX	416	Property_z5_31	531	D_1_AC_LAG_8_MIN	646	D_0:2_CC_LAG_2_MAX
72	D_4:0_CC_LAG_6_MAX	187	D_0:3_CC_LAG_5_MIN	302	D_1:2_CC_LAG_2_MIN	417	D_4:1_CC_LAG_6_MIN	532	Property_z1_30	647	D_2_AC_LAG_9_MAX
73	D_0:3_CC_LAG_2_MAX	188	D_4:3_CC_LAG_8_MAX	303	D_4:0_CC_LAG_9_MAX	418	D_4:2_CC_LAG_7_MAX	533	D_2:0_CC_LAG_6_MAX	648	Property_z2_27
74	Property_z4_8	189	D_1:4_CC_LAG_5_MAX	304	D_2:0_CC_LAG_4_MIN	419	D_3:1_CC_LAG_8_MAX	534	Property_z2_17	649	Property_z1_27
75	Property_z1_6	190	Property_z5_13	305	D_0:4_CC_LAG_4_MIN	420	D_1:3_CC_LAG_9_MIN	535	D_3:2_CC_LAG_9_MIN	650	D_2_AC_LAG_5_MIN
76	Property_z5_5	191	D_0:4_CC_LAG_0_MAX	306	D_0_AC_LAG_0_MAX	421	D_3:4_CC_LAG_9_MAX	536	D_1:2_CC_LAG_7_MAX	651	D_2_AC_LAG_3_MAX
77	D_3:0_CC_LAG_1_MIN	192	D_1:3_CC_LAG_0_MIN	307	D_3_AC_LAG_1_MIN	422	D_1:3_CC_LAG_1_MAX	537	D_3_AC_LAG_9_MIN	652	D_2_AC_LAG_5_MAX
78	Property_z1_0	193	Property_z1_33	308	D_0:3_CC_LAG_6_MIN	423	D_2:1_CC_LAG_5_MAX	538	D_2:3_CC_LAG_7_MAX	653	Property_z1_26
79	D_4_AC_LAG_4_MIN	194	D_0:3_CC_LAG_3_MAX	309	D_2:4_CC_LAG_5_MAX	424	D_0:1_CC_LAG_7_MAX	539	D_2:3_CC_LAG_1_MIN	654	D_2_AC_LAG_0_MAX
80	Property_z4_3	195	D_1:0_CC_LAG_7_MIN	310	D_1:4_CC_LAG_6_MAX	425	D_0:4_CC_LAG_8_MIN	540	D_2:4_CC_LAG_7_MAX	655	Property_z4_26
81	D_0:4_CC_LAG_2_MIN	196	D_4:1_CC_LAG_3_MAX	311	D_1:3_CC_LAG_4_MIN	426	D_1:2_CC_LAG_4_MAX	541	Property_z5_19	656	D_2_AC_LAG_3_MIN
82	D_1:4_CC_LAG_4_MAX	197	D_4:0_CC_LAG_2_MAX	312	D_0:3_CC_LAG_7_MAX	427	D_0_AC_LAG_9_MIN	542	D_1:0_CC_LAG_9_MAX	657	D_2_AC_LAG_0_MIN
83	Property_z5_4	198	D_4:0_CC_LAG_0_MIN	313	D_0:2_CC_LAG_7_MIN	428	D_3_AC_LAG_1_MAX	543	D_4:3_CC_LAG_0_MIN	658	Property_z5_26
84	Property_z1_34	199	D_2:0_CC_LAG_3_MIN	314	D_0_AC_LAG_2_MAX	429	D_4:1_CC_LAG_4_MIN	544	D_3:2_CC_LAG_2_MIN	659	D_2_AC_LAG_4_MAX
85	Property_z1_14	200	Property_z1_16	315	D_4_AC_LAG_9_MIN	430	D_1_AC_LAG_4_MAX	545	Property_z3_27	660	Property_z4_18
86	NetCharge@9.0	201	D_0:3_CC_LAG_0_MAX	316	D_0:1_CC_LAG_1_MAX	431	D_2:0_CC_LAG_9_MIN	546	D_2:1_CC_LAG_2_MAX	661	D_2_AC_LAG_4_MIN
87	Property_z5_3	202	D_4:1_CC_LAG_3_MIN	317	D_3:4_CC_LAG_6_MIN	432	D_3:1_CC_LAG_7_MAX	547	D_2:3_CC_LAG_2_MIN	662	Property_z2_26
88	D_1:0_CC_LAG_0_MIN	203	D_4_AC_LAG_9_MAX	318	D_1:0_CC_LAG_9_MIN	433	Property_z4_31	548	D_2:0_CC_LAG_5_MAX	663	D_2_AC_LAG_6_MIN
89	D_3:0_CC_LAG_2_MAX	204	D_0_AC_LAG_6_MAX	319	D_3_AC_LAG_6_MAX	434	D_4:2_CC_LAG_2_MAX	549	Property_z3_19	664	D_2_AC_LAG_6_MAX
90	Property_z3_11	205	D_0:1_CC_LAG_6_MIN	320	D_3:1_CC_LAG_6_MIN	435	D_1:0_CC_LAG_5_MAX	550	D_3_AC_LAG_2_MIN	665	Property_z1_24
91	Property_z1_10	206	D_4:0_CC_LAG_5_MIN	321	Property_z3_32	436	D_0:1_CC_LAG_6_MAX	551	D_1:2_CC_LAG_9_MAX	666	D_2_AC_LAG_8_MIN
92	Property_z5_0	207	D_2:1_CC_LAG_1_MIN	322	D_1:0_CC_LAG_3_MAX	437	D_4:1_CC_LAG_5_MIN	552	Property_z3_30	667	Property_z4_25
93	D_4:0_CC_LAG_3_MAX	208	D_4:1_CC_LAG_4_MAX	323	D_2:1_CC_LAG_5_MIN	438	D_1_AC_LAG_3_MAX	553	D_3:4_CC_LAG_7_MIN	668	Property_z4_19
94	D_0:3_CC_LAG_0_MIN	209	D_2:4_CC_LAG_3_MAX	324	D_4:3_CC_LAG_9_MAX	439	Property_z3_17	554	D_2:3_CC_LAG_4_MIN	669	D_2_AC_LAG_7_MAX

95	Property_z3_6	210	D_3:0_CC_LAG_3_MIN	325	D_1:4_CC_LAG_7_MIN	440	D_3_AC_LAG_6_MIN	555	D_3_AC_LAG_9_MAX	670	Property_z5_25
96	D_1:4_CC_LAG_0_MAX	211	D_1:2_CC_LAG_5_MIN	326	D_0:1_CC_LAG_8_MIN	441	D_0_AC_LAG_7_MIN	556	Property_z1_21	671	D_2_AC_LAG_8_MAX
97	D_0:1_CC_LAG_3_MIN	212	Property_z1_15	327	D_0:1_CC_LAG_4_MAX	442	D_3:2_CC_LAG_2_MAX	557	D_0:2_CC_LAG_3_MAX	672	D_2_AC_LAG_7_MIN
98	Property_z1_7	213	Isoelectric point	328	D_3:4_CC_LAG_8_MAX	443	D_0:1_CC_LAG_9_MAX	558	D_4:2_CC_LAG_7_MIN	673	Property_z2_25
99	D_0:4_CC_LAG_6_MAX	214	D_3:4_CC_LAG_4_MAX	329	D_2:0_CC_LAG_8_MAX	444	D_1:4_CC_LAG_9_MIN	559	D_2:1_CC_LAG_1_MAX	674	D_2_AC_LAG_1_MAX
100	D_0:4_CC_LAG_0_MIN	215	D_0:1_CC_LAG_2_MIN	330	D_2:0_CC_LAG_5_MIN	445	D_3:1_CC_LAG_4_MAX	560	D_3:0_CC_LAG_9_MAX	675	Property_z4_24
101	Property_z3_15	216	D_4:0_CC_LAG_8_MAX	331	D_0:3_CC_LAG_6_MAX	446	D_3:2_CC_LAG_1_MIN	561	D_4:3_CC_LAG_9_MIN	676	D_2_AC_LAG_2_MAX
102	D_1:2_CC_LAG_4_MIN	217	D_1_AC_LAG_7_MAX	332	D_1_AC_LAG_0_MIN	447	D_2:0_CC_LAG_4_MAX	562	D_4:2_CC_LAG_3_MIN	677	Property_z4_20
103	D_0:4_CC_LAG_5_MAX	218	D_1:3_CC_LAG_2_MIN	333	D_3:1_CC_LAG_5_MIN	448	D_1:0_CC_LAG_8_MAX	563	D_3:2_CC_LAG_1_MAX	678	Property_z5_24
104	Property_z4_13	219	Property_z3_33	334	D_1:2_CC_LAG_6_MAX	449	Property_z3_31	564	Property_z5_17	679	D_2_AC_LAG_1_MIN
105	Property_z3_4	220	D_1:0_CC_LAG_1_MAX	335	D_1_AC_LAG_2_MAX	450	D_3_AC_LAG_5_MIN	565	D_1:2_CC_LAG_3_MAX	680	D_2_AC_LAG_2_MIN
106	D_4:1_CC_LAG_2_MAX	221	D_4:3_CC_LAG_6_MAX	336	Property_z2_15	451	D_2:1_CC_LAG_8_MIN	566	Property_z5_29	681	Property_z5_23
107	Property_z2_33	222	Property_z4_14	337	D_0_AC_LAG_9_MAX	452	D_2:4_CC_LAG_8_MAX	567	D_2:3_CC_LAG_6_MAX	682	Property_z4_23
108	D_0:3_CC_LAG_1_MIN	223	D_1:4_CC_LAG_3_MAX	338	D_3_AC_LAG_5_MAX	453	D_3:1_CC_LAG_3_MAX	568	D_4:2_CC_LAG_4_MIN	683	Property_z1_25
109	D_4:1_CC_LAG_0_MIN	224	D_0_AC_LAG_5_MAX	339	D_1:4_CC_LAG_2_MIN	454	Wimley-White Partitioning (pH7.0)	569	D_2:4_CC_LAG_6_MIN	684	Property_z4_21
110	D_4_AC_LAG_6_MIN	225	D_3:0_CC_LAG_7_MIN	340	Property_z2_32	455	D_3_AC_LAG_0_MAX	570	D_0:2_CC_LAG_4_MAX	685	Property_z4_22
111	D_0:2_CC_LAG_1_MIN	226	D_4_AC_LAG_5_MAX	341	D_1:2_CC_LAG_1_MIN	456	D_4:1_CC_LAG_9_MIN	571	Property_z5_20	686	Molecular Weight
112	D_4:3_CC_LAG_5_MAX	227	D_4:3_CC_LAG_1_MAX	342	D_4:2_CC_LAG_4_MAX	457	D_4:2_CC_LAG_1_MAX	572	D_2:3_CC_LAG_5_MAX	687	Property_z5_22
113	D_1:0_CC_LAG_6_MIN	228	D_2:1_CC_LAG_0_MAX	343	D_3:0_CC_LAG_9_MIN	458	D_1:3_CC_LAG_6_MAX	573	D_2:4_CC_LAG_9_MIN	688	Size
114	D_0_AC_LAG_0_MIN	229	D_3:1_CC_LAG_0_MIN	344	D_3:0_CC_LAG_4_MAX	459	Property_z3_29	574	D_0:2_CC_LAG_9_MAX	689	Property_z5_21
115	Property_z3_5	230	D_4:1_CC_LAG_7_MAX	345	D_0:3_CC_LAG_7_MIN	460	D_3:2_CC_LAG_3_MIN	575	Property_z4_29		
116	D_3:4_CC_LAG_3_MAX	231	D_3:4_CC_LAG_6_MAX	346	D_0:3_CC_LAG_5_MAX	461	Property_z4_15	576	D_4:2_CC_LAG_2_MIN		

2.2 Figures

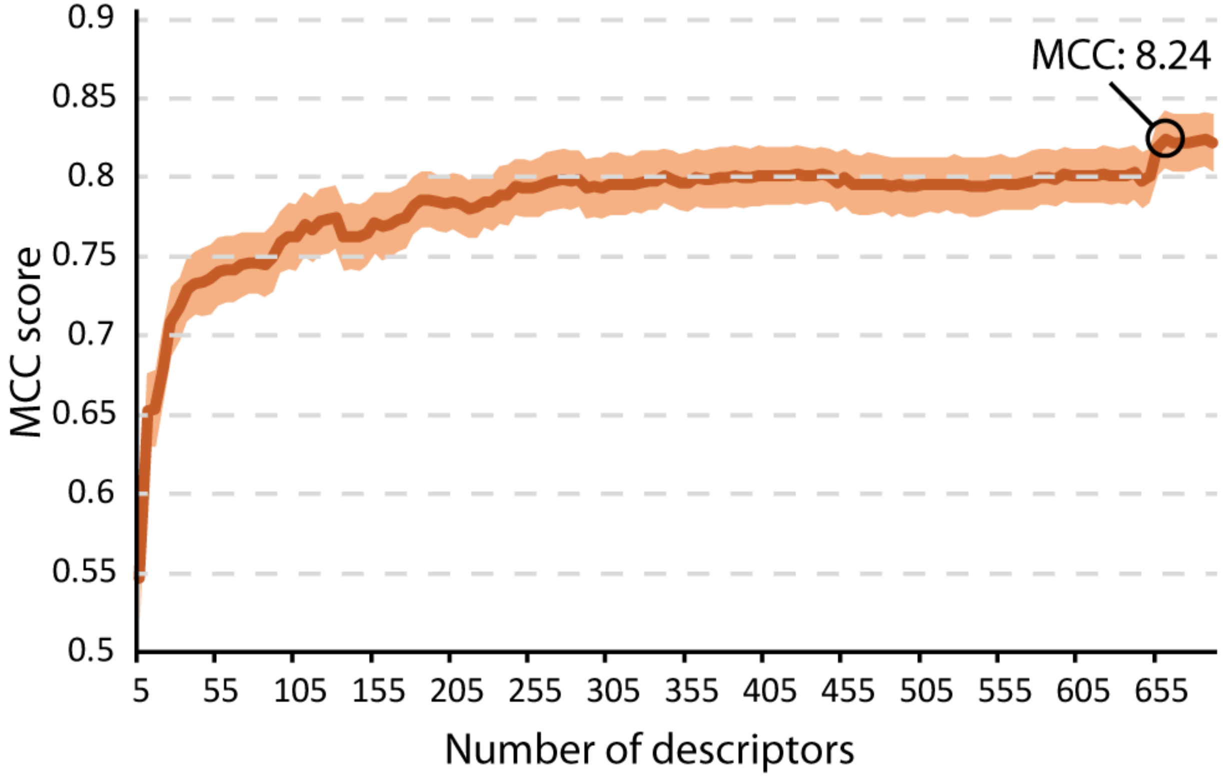

Figure S1. IFS results. Ten-fold cross validation of the sorted list of descriptors. The descriptor list was sorted by Maximum Relevance, Minimum Reduncancy (mRMR) and a total number of 138 models were trained. The model giving the highest MCC score was selected.
